# Insights into enterotoxigenic *Escherichia coli* diversity in Bangladesh utilizing genomic epidemiology

**DOI:** 10.1038/s41598-017-03631-x

**Published:** 2017-06-13

**Authors:** Jason W. Sahl, Jeticia R. Sistrunk, Nabilah Ibnat Baby, Yasmin Begum, Qingwei Luo, Alaullah Sheikh, Firdausi Qadri, James M. Fleckenstein, David A. Rasko

**Affiliations:** 1 Institute for Genome Sciences, Department of Microbiology and Immunology, University of Maryland School of Medicine, 801 W. Baltimore Street, Suite 600, Baltimore, MD 21201 USA; 2 Translational Genomics Research Institute, Flagstaff, AZ 86001 USA; 3 Centre for Vaccine Sciences, Immunology Laboratory, International Centre Center for Diarrhoeal Disease Research, Mohakhali, Dhaka, 1212 Bangladesh; 4 Department of Medicine, Division of Infectious Diseases, Washington, USA; 5 The Molecular Microbiology and Microbial Pathogenesis Program, Division of Biology and Biomedical Sciences, Washington University in St. Louis, Washington, USA; 6 Medicine Service, Veterans Affairs Medical Center, St. Louis, MO USA

## Abstract

Enterotoxigenic *Escherichia coli* (ETEC) cause more than 500,000 deaths each year in the developing world and are characterized on a molecular level by the presence of genes that encode the heat-stable (ST) and/or heat-labile (LT) enterotoxins, as well as surface structures, known as colonization factors (CFs). Genome sequencing and comparative genomic analyses of 94 previously uncharacterized ETEC isolates demonstrated remarkable genomic diversity, with 28 distinct sequence types identified in three phylogenomic groups. Interestingly, there is a correlation between the genomic sequence type and virulence factor profiles based on prevalence of the isolate, suggesting that there is an optimal combination of genetic factors required for survival, virulence and transmission in the most successful clones. A large-scale BLAST score ratio (LS-BSR) analysis was further applied to identify ETEC-specific genomic regions when compared to non-ETEC genomes, as well as genes that are more associated with clinical presentations or other genotypic markers. Of the strains examined, 21 of 94 ETEC isolates lacked any previously identified CF. Homology searches with the structural subunits of known CFs identified 6 new putative CF variants. These studies provide a roadmap to exploit genomic analyses by directing investigations of pathogenesis, virulence regulation and vaccine development.

## Introduction

The enterotoxigenic *Escherichia coli* (ETEC) pathogenic variant (pathovar) has been implicated in 1 billion cases of diarrhea annually^1–3^. These pathogens are especially problematic in ETEC endemic areas, such as Bangladesh^4, 5^. ETEC are characterized on a molecular basis by the presence of genes that encode the heat-stable (ST) and/or heat-labile (LT) enterotoxin^6, 7^. Both toxins activate the cystic fibrosis trans-membrane regulator (CFTR) that results in ion secretion followed by water and diarrhea in infected individuals^8^. In addition to the enterotoxins, ETEC possess fimbrial appendages that attach to intestinal epithelium known as colonization factors (CFs). Most ETEC-specific virulence factors including the CFs are plasmid-encoded, with greater than 30 CFs described in the literature^9, 10^.

In addition to known ETEC virulence factors, other putative virulence factors have been identified, primarily in the prototypical ETEC isolate, H10407^11^. These factors include the adhesin autotransporter TibA^12^, the invasion locus *tia* ^13^, and *leoA* ^14^, which has been associated with maximum LT secretion. Additionally, the serine protease autotransporter EatA^15^, which has only been identified in ETEC isolates^16^, accelerates delivery of LT by degrading MUC2, the major mucin secreted by gastrointestinal goblet cells^17^, and by modulating adhesion mediated by EtpA^18^. EtpA is a glycoprotein that appears to act as a bridge between FliC and host surface structures^19, 20^. In addition to these previously identified ETEC-associated factors, a study by Vidal *et al*.^21^ suggested that an iron acquisition system (*irp2*, *fyuA*) most similar to a system in *Yersinia* species^22, 23^ may play a role in ETEC virulence based on its variable presence in clinical ETEC isolates^24^.

The majority of the functional work in ETEC has focused on the prototype isolate H10407^25, 26^. However, detailed transcriptional studies using RNA-seq have demonstrated that the response to environmental and host signals, such as bile and carbohydrates, can vary widely between ETEC isolates^27^. Additionally, it had been suggested that some putative virulence factors identified in H10407 are not widely distributed among diverse ETEC isolates^28^. This suggests that the genomic diversity within ETEC isolates is significant and a reference-independent global approach is required to comprehensively characterize the genomic diversity.

The advent of large-scale sequencing has increased our understanding of the evolution of the members of the ETEC pathovar. Until 2014, there were relatively few sequenced and assembled human-associated ETEC isolates, all from symptomatic patients, available in Genbank^11, 16, 29^, as well as, four porcine ETEC isolates that had also been sequenced^30^. A recent study by von Mentzer *et al*. in 2014 utilized a genomic mapping approach of unassembled genomes for the examination of genome similarity in a collection of 362 global *E. coli* isolates^31^. The isolates sequenced in the von Mentzer *et al*. study were selected for the greatest variability of colonization factor and enterotoxin profiles from a historical collection ranging from 1980 and 2011 of ETEC isolates maintained at the University of Gothenburg. The current study examines a collection of 94 ETEC isolates, 89 of which represent circulating isolates within Bangladesh between 2002 and 2011. Additionally, 84 isolates were obtained from individuals with diarrhea (symptomatic ETEC) and 10 isolates obtained from asymptomatic ETEC colonization. Comparative genomics of this wealth of information is providing novel insights into the evolution and distribution of ETEC virulence factors.

Although large-scale sequencing projects can now rapidly generate a data from large numbers of isolates, informatics pipelines and comparative analyses to take advantage of these large-scale genomic data have languished. These types of genomic epidemiology studies have been recently completed with other *E. coli* pathovars^32–34^, but this study provides a further example of the application of this comparative analysis paradigm to isolates from the ETEC pathovar.

## Results

### Core genome single nucleotide polymorphism (SNP) phylogeny of ETEC

**Figure 1:**
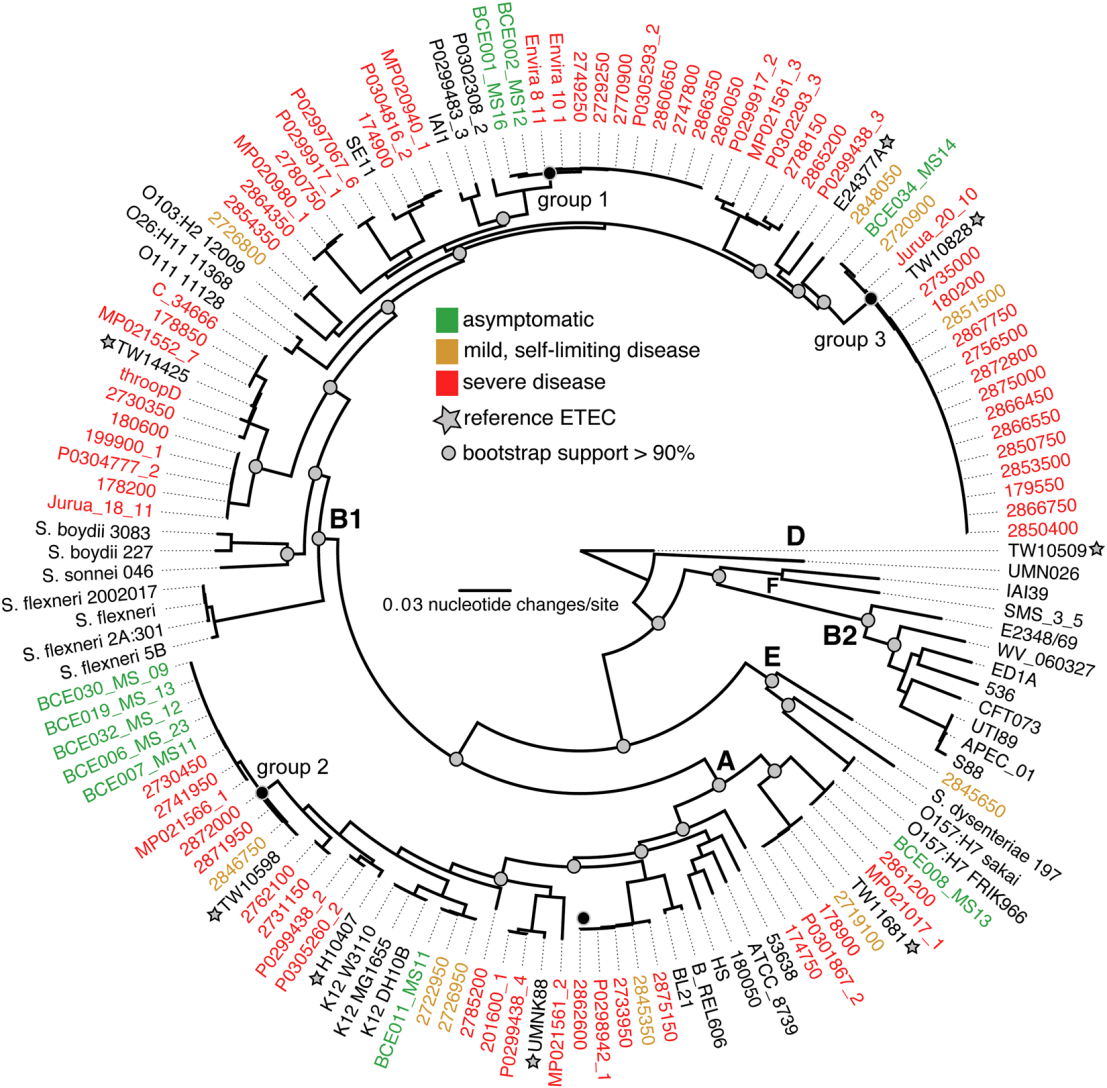
A core genome single nucleotide polymorphism (SNP) phylogeny of ETEC genomes sequenced in this study as well as reference *E. coli* genomes. SNPs were identified by NUCmer^59^ alignments of query genomes against the reference genome, K-12 W3110; these methods were wrapped by the NASP pipeline^62^. A phylogeny was inferred on the concatenated SNP alignment using RAxML v8^60^ including 100 bootstrap replicates. ETEC genomes sequenced in this study were assigned to disease groups based on clinical observations at site of isolation or presented in literature.

To examine the phylogenetic relationship of the sequenced ETEC isolates in the broader context of diverse *E. coli* and *Shigella* spp., a SNP-based phylogeny was inferred from ~220,000 SNPs from 136 *E. coli/Shigella* genomes (Supplementary Data File 1). The genomes from this study include the 94 ETEC genomes sequenced in this study, eight previously sequenced ETEC reference genomes, 34 reference *E. coli* and *Shigella* genomes representing prototype members of each of the diarrheagenic pathogenic variants (Metadata and GenBank Accession numbers are included in Table S1). The results demonstrate that the majority of ETEC genomes fall into the *E. coli* phylogroups A or B1 (Fig. 1), with one genome, isolate 2845650, falling into phylogroup E; ETEC isolates from this phylogroup have been previously described^31^. The retention index (RI) value of 0.82 for this tree was determined using Phangorn, suggesting significant homoplasy, likely resulting from homologous recombination.

The phylogenetic diversity of sequenced ETEC isolates was remarkable (Fig. 1). The majority of the diarrheagenic ETEC (symptomatic ETEC) sequenced in this study were isolated from Dhaka, Bangladesh between 2002 and 2011, yet based on the relatedness displayed in the phylogeny, the isolates are broadly distributed across the known diversity in *E. coli*. This highlights that the genetic background of *E. coli* is generally amenable to the uptake and maintenance of ETEC plasmids and virulence factors. However, no currently sequenced ETEC genomes, of the ~450 ETEC isolates, are present within Phylogroup B2, which suggests that this particular genetic background is not amenable for the uptake or retention of ETEC plasmids. The study by von Mentzer *et al*.^31^ suggested that three B2 ETEC genomes were identified however, one of the reported B2 genomes (E1642) was not B2 by our phylogenomic analysis (Figure S1), and the other two (E523, E2439) were negative for all queried ETEC enterotoxins (0% toxin coverage at a minimum depth of 2x), indicating that they were not ETEC based on the sequence data that is publically available.

Despite this overall phylogenetic diversity, strains isolated from geographically and temporally dispersed cases of cholera-like illness were phylogenetically similar. These results taken as a whole indicate that particular combinations of pathovar-specific genes and genomic backgrounds may be optimal for survival, virulence and transmission as determined by the prevalence of the combinations in these and other studies^31^; however, detailed transcriptional studies are required for this to be elucidated.

### Bioinformatic analysis of symptomatic ETEC and asymptomatic ETEC for known and putative virulence factors

**Figure 2:**
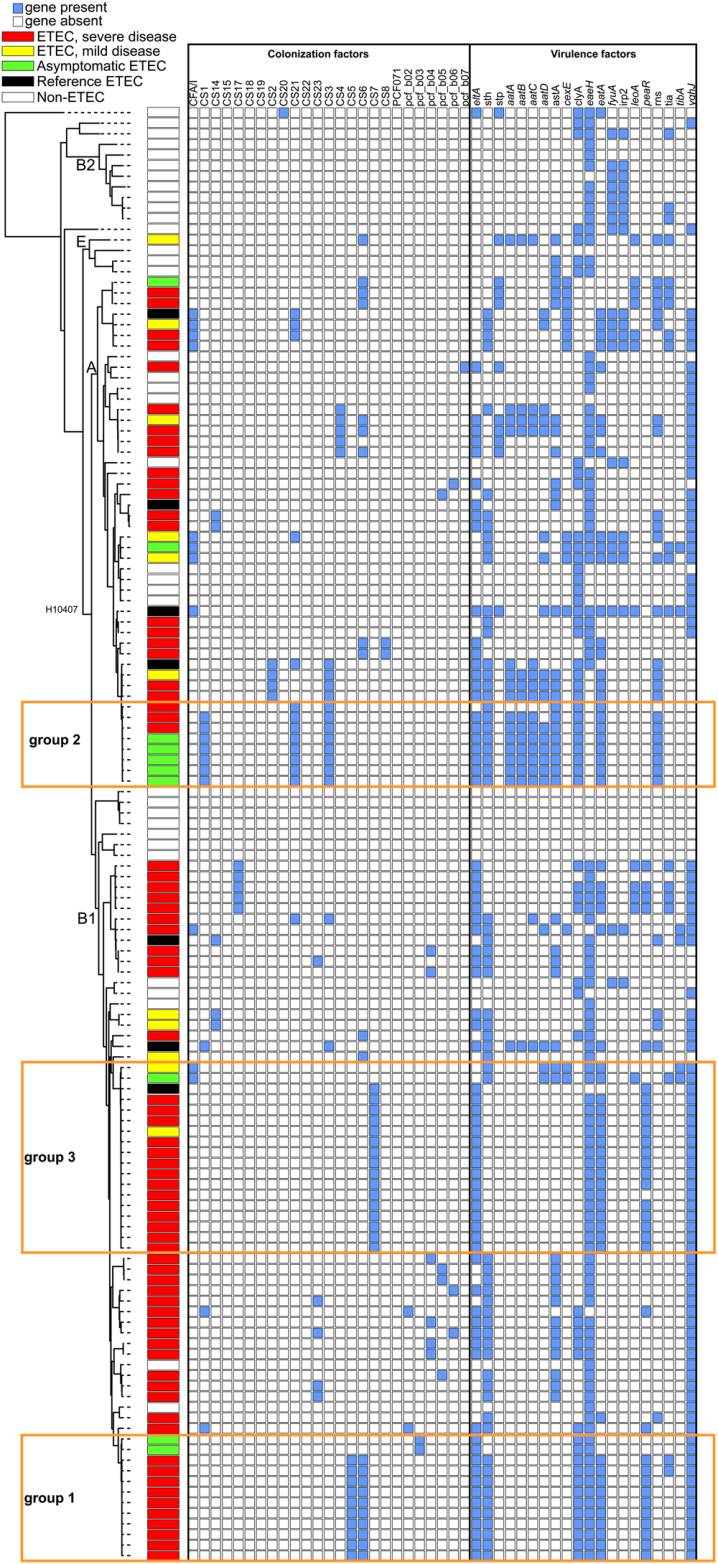
A core genome single nucleotide polymorphism (SNP) phylogeny of ETEC genomes sequenced in this study as well as reference *E. coli* genomes associated with a heatmap of BSR values of previously-characterized virulence and colonization factors (Table S3). Disease categories were assigned based on clinical observations. Orange brackets around genomes indicate lineages (Groups 1–3) compared to identify coding regions associated with the observed clinical presentations. The heatmap was associated with the phylogeny using the interactive tree of life (47).

The ETEC isolates sequenced in this study were identified as members of this pathotype, as they contained one or more of the enterotoxin genes (LT, STh and/or STp) (Fig. 2, Table S3). The presence of additional known and predicted virulence factor genes was determined using LS-BSR^35^. The results demonstrate that the previously identified virulence factors of ETEC do not cluster among either the asymptomatic ETEC or symptomatic ETEC isolates (Fig. 2). Many of the virulence factors (e.g. *leoA*) identified in the prototype isolate H10407^11^ were sparsely distributed among the 94 isolates sequenced in this study (Fig. 2). The *etpA* gene was included in the initial analyses, but the five repeat regions in the 3′ end of the gene, each ~600 nucleotides, confounded proper assembly of this genomic region and precluded an accurate estimation of *etpA* conservation. Proteomic analysis^36^ of the strains corresponding to the genomes analyzed here found that EtpA was produced by more than 60% of the isolates, and that the sizes of the secreted peptides were similar to that reported for H10407 (~170 kD). Thus, *etpA* was removed from downstream *in silico* analyses.

In examining correlations between individual known and predicted virulence factors and the corresponding clinical presentation associated with the respective isolate, no single virulence factor or gene region segregated exclusively with symptomatology or phylogenomic group (Fig. 2). Thus, there is not a single proposed virulence factor that can conclusively and consistently distinguish ETEC isolates that are diarrhea-associated or only from an asymptomatic colonization.

The data analysis did reveal correlations between certain combinations of virulence factors, CF types, and phylogenomic location suggesting that some strains may possess a suite of features that are more optimal for human infection. The boxes highlighted in Fig. 2 demonstrate phylogenetic groups of isolates that have similar genomic content as defined by the phylogeny, and similar virulence factor profiles. As highlighted in Fig. 2, there are three monophyletic groups with a significant number of isolates that have similar virulence and colonization factor profiles, but are variable for the clinical presentations. These groups are labeled groups 1–3 in this study and can be correlated, but not perfectly matched to the previously identified dominant lineages 5, 1/2 and 3, respectively from von Mentzer *et al*.^31^. The isolates in Group 1 are dominated by symptomatic ETEC isolates, the majority of which contain CS5 and CS6 (Figure S2). In contrast, the asymptomatic isolates from this study in this phylogenomic group are only positive for a novel CF identified in the current study (see below). The distinction in this phylogenomic group based on clinical presentation is extended to the virulence factors, as all the symptomatic isolates are generally LT and ST positive (22/29 isolates), whereas the asymptomatic isolates are only LT positive (Figure S2). Interestingly, the symptomatic isolates in this phylogenomic cluster are also positive for the regulator known as *peaR* ^27^, which has homology to the *rns* regulator which is involved in colonization factor regulation^37^. Group 2 isolates possess genes for CS1, CS3 and CS21 (Figure S2). Group 2 isolates contain both symptomatic ETEC and asymptomatic ETEC isolates (13 versus 7, respectively). One of the largest phylogenetic groups, Group 3 in Figs 1 and 2, consisted primarily of isolates from symptomatic infections, with only one isolate obtained from asymptomatic colonization. This group contains primarily colonization factors CFA/I or CS7, and encoded LT and ST genes. Overall, these examples demonstrate that there is significant genomic and virulence factor diversity among these isolates, but there are also common combinations of virulence factors and genomic backgrounds that may create a more optimal pathogen or allow the isolate to survive in the environment. These more common clones will need to be examined functionally, via detailed mutagenesis, transcriptional and virulence assays to test these hypotheses.

### Genomic epidemiology comparison of symptomatic ETEC and asymptomatic ETEC genome content

To identify any genomic regions unique to symptomatic ETEC isolates when compared to asymptomatic ETEC isolates, a whole-genome large-scale blast score ratio (LS-BSR)^35^ comparison was conducted on the ETEC genomes sequenced in this study as well as a set of previously sequenced ETEC genomes (Table S1). The results demonstrate that no coding regions (CDSs) were exclusive to either asymptomatic ETEC (n = 23) or symptomatic ETEC (n = 262).

While no features could be identified that were exclusive in each of the clinical presentations, a statistical approach identified genomic regions that were associated with either the asymptomatic ETEC or symptomatic ETEC. The LS-BSR data set generated here (Supplementary Data File 2) was examined for the prevalence of gene features with either the clinical symptomology or phylogenetic distribution. When the genomes were examined for features that were associated with symptomatic ETEC or asymptomatic ETEC, we identified 28 features that were statistically (FDR p value < 0.05) associated with symptomatic ETEC (Table S4) and 28 features statistically associated with the asymptomatic ETEC (Table S4). These features highlight the use of genomic epidemiology for the identification of features associated with virulence. The genes associated with symptomatic ETEC isolates included a number of hypothetical proteins, phage related genes, as well as transcriptional regulators (Table S4). Coding regions that were associated with the asymptomatic isolates primarily included hypothetical proteins (Table S4). While none of these features stand out as obvious virulence factors, and do not demonstrate a difference in prevalence between ETEC and other pathovar isolates, they do provide additional evidence of features that may play a role in the interactions of the host and pathogen. These *in silico* studies provide a starting point for the functional analysis of these genes and the potential role they play in virulence.

Our group has previously identified that there is a component of the ETEC genomes is phylogenomically linked^16, 27^. While these earlier calculations were based on far fewer genomes, the general trends of those studies are confirmed in this study, as the majority of ETEC isolates were identified in phylogroups A or B1 (Fig. 1), with only one ETEC genome in this study identified in phylogroup E. As with the clinical presentation comparisons, we do not observe canonical virulence factors in these groups, but rather a number of genes that could serve as accessory functions to virulence.

In addition to a global comparison of all symptomatic ETEC and asymptomatic ETEC, we also attempted to identify specific regions unique to symptomatic ETEC in two lineages (group 1 and 2) of the inferred phylogeny (Figures 1 and 2), chosen due to the substantial ETEC representation on these lineages (Figure S2). This analysis was performed to identify genomic regions that could explain differences in clinical presentation among patients colonized with phylogenetically-related isolates. Genomic regions were identified in these two clades that could largely differentiate the asymptomatic ETEC and symptomatic ETEC isolates (Table 1). The asymptomatic ETEC and symptomatic ETEC in group 2 contained 16 and 9 coding regions, respectively, that could be used to distinguish isolates based on clinical presentation on this branch (Supplementary Data File 3). Many of these features are associated with plasmids (Table S4), suggesting that divergent plasmids, or parts of plasmids, may have been acquired by the isolates in these two different groups. However, because the isolates were not collected contemporaneously, (Table S1), we cannot exclude temporal effects on genome content and it remains unclear whether these regions are explicitly associated with pathogenesis.

**Table 1:** Features associated with clinical presentation or phylogenomic groupings.

Condition	FDR p value < 0.05
Symptomatic	11
Asymptomatic	26
Group 1 symptomatic	7
Group 1 asymptomatic	7
Group 2 symptomatic	0
Group 2 asymptomatic	0

No single gene feature distinguished ETEC associated with symptomatic illness or asymptomatic colonization. However comparative analyses within phylogenetic groups did identify genes that statistically segregate with the clinical presentation, thus providing stable genomic encoded targets for virulence studies, functional characterization and/or additional phylogenomic features for use as diagnostic markers, as we have previously done in other *E. coli* pathovars^38, 39^.

### Comparison of ETEC and non-ETEC genomes

**Figure 3:**
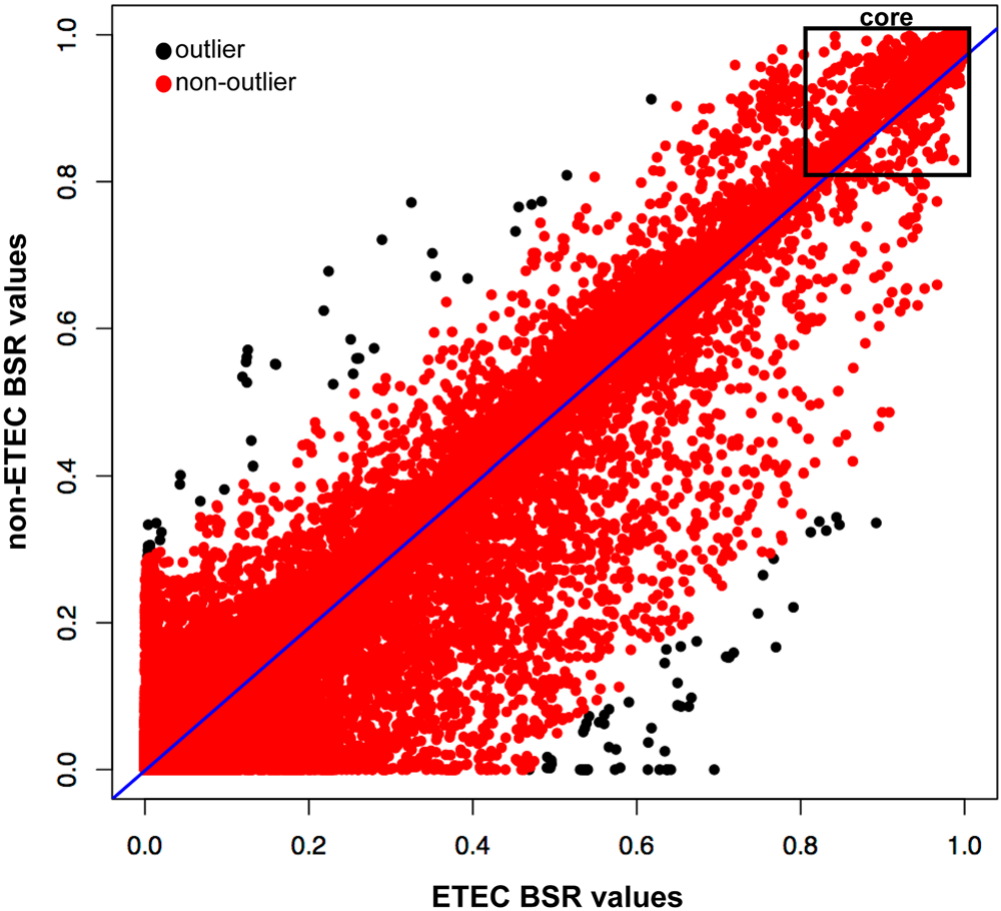
A comparison of BSR values^69^ between ETEC (n = 253) and non-ETEC (n = 253) in phylogroups A and B1. A total of 118 genes that are outliers are identified and shown in black as defined by the MASS package in R. A functional breakdown of these genes is listed in Table S4.

A previous study of *E. coli* comparative genomics using far fewer genomes, demonstrated pathovar-specific genome conservation among ETEC isolates^16^. The earlier study utilized a reference-based method that included only seven symptomatic ETEC genomes, a relatively small number of genomes compared to those analyzed in this study and the von Mentzer study^31^. Using a global approach, the genomic content of ETEC and non-ETEC isolates was examined in *E. coli* phylogroups A and B1, where the majority of ETEC isolates are located. A total of 506 genomes from phylogroups A and B1 (253 ETEC and 253 non-ETEC; Table S2) were compared in a LS-BSR analysis. While most coding regions were distributed between groups, outliers (n = 118) were identified (Table 2, Table S4; Fig. 3). Several of the ETEC-specific regions were associated with the ETEC toxins and putative plasmid components, as expected (Table S4). However, the non-ETEC genomes contained coding regions with various functional annotations, including features of central metabolism and type III secretion genes, suggesting that a specific genomic background and selective pressures are involved in the acquisition and retention of ETEC plasmids that harbor enterotoxins, as well as the non-ETEC virulence factors.

**Table 2:** Top 20 genes Identified as ETEC or non-ETEC specific.

Centroid ID	annotation^a^	Average BSR (ETEC)	Average BSR (non-ETEC)
centroid_109500	methyltransferase small domain protein	**0.831225296**	0.325454545
centroid_185863	putative plasmid maintenance protein	**0.769881423**	0.166758893
centroid_491002	type IV secretion protein Rhs	**0.767035573**	0.287193676
centroid_286834	heat-stable enterotoxin	**0.69458498**	0
centroid_401212	plasmid segregation protein ParM	**0.673162055**	0.174703557
centroid_352933	hypothetical protein pEntH10407_p04	**0.666679842**	0.097905138
centroid_2111416	diguanylate cyclase domain protein	**0.654031621**	0.086007905
centroid_1195229	heat-labile enterotoxin subunit A	**0.641581028**	0
centroid_1149275	heat-labile enterotoxin subunit A	**0.637114625**	0
centroid_976050	heat-labile enterotoxin B chain	**0.636600791**	0
centroid_584031	protein StbB	**0.636086957**	0.163754941
centroid_146011	serine protease EatA	**0.6343083**	0.025059289
centroid_957787	insA N-terminal domain protein	**0.590434783**	0.091857708
centroid_174198	CFA/I fimbrial subunit D	**0.579762846**	0.002648221
centroid_1146026	plasmid stability family protein	**0.574664032**	0.027549407
centroid_31447	plasmid segregation protein ParM	**0.566086957**	0.030671937
centroid_208934	putative transposase domain protein	**0.541857708**	0.072687747
centroid_33993	POTRA domain, ShlB-type family protein	**0.535652174**	0
centroid_372954	heat-stable enterotoxin	**0.530948617**	0
centroid_740634	putative transporter protein AatB	**0.494387352**	0.002173913
centroid_1853128	bacterial type II/III secretion system short domain protein	0.003280632	**0.29083004**
centroid_1844289	LEE encoded regulator	0.003596838	**0.298695652**
centroid_1836929	type III secretion apparatus protein, YscR/HrcR family	0.003952569	**0.293399209**
centroid_1405762	type III secretion apparatus protein SpaR/YscT/HrcT	0.003952569	**0.28972332**
centroid_1742164	type III secretion, HrpO family protein	0.003952569	**0.292648221**
centroid_1726625	tir chaperone	0.003952569	**0.29826087**
centroid_1827912	type III secretion effector delivery regulator, TyeA family	0.003952569	**0.289328063**
centroid_1613860	type III secretion system regulator family protein	0.003952569	**0.304505929**
centroid_1754850	type III secretion apparatus needle protein	0.003952569	**0.304347826**
centroid_1761547	type III secretion low calcium response chaperone LcrH/SycD	0.003952569	**0.290395257**
centroid_1737915	secretion system apparatus protein SsaV	0.004466403	**0.291304348**
centroid_1228577	N(2)-citryl-N(6)-acetyl-N(6)-hydroxylysine synthase	0.005098814	**0.294071146**
centroid_1614162	aerobactin synthase	0.00541502	**0.296363636**
centroid_1222308	N(6)-hydroxylysine O-acetyltransferase	0.007351779	**0.295731225**
centroid_1399399	serine/threonine-protein phosphatase	0.067549407	**0.365573123**
centroid_818055	calcineurin-like phosphoesterase superfamily domain protein	0.229644269	**0.524229249**
centroid_676376	gnsA/GnsB family protein	0.261304348	**0.559407115**
centroid_1832824	cold shock protein CspA	0.350632411	**0.702490119**
centroid_269217	cold shock-like protein CspG	0.354743083	**0.671185771**
centroid_408267	L-fucose-proton symporter domain protein	0.617747036	**0.91229249**

^a^Genes with annotation of hypothetical or conserved hypothetical have been removed from the table. The complete gene list is present in Supplemental Table S4.

The bold values included in the table highlights which genes have an average LS-BSR suggesting ETEC (top of table) or non-ETEC (bottom of table) prevalence.

### *In silico* colonization factor identification

**Figure 4:**
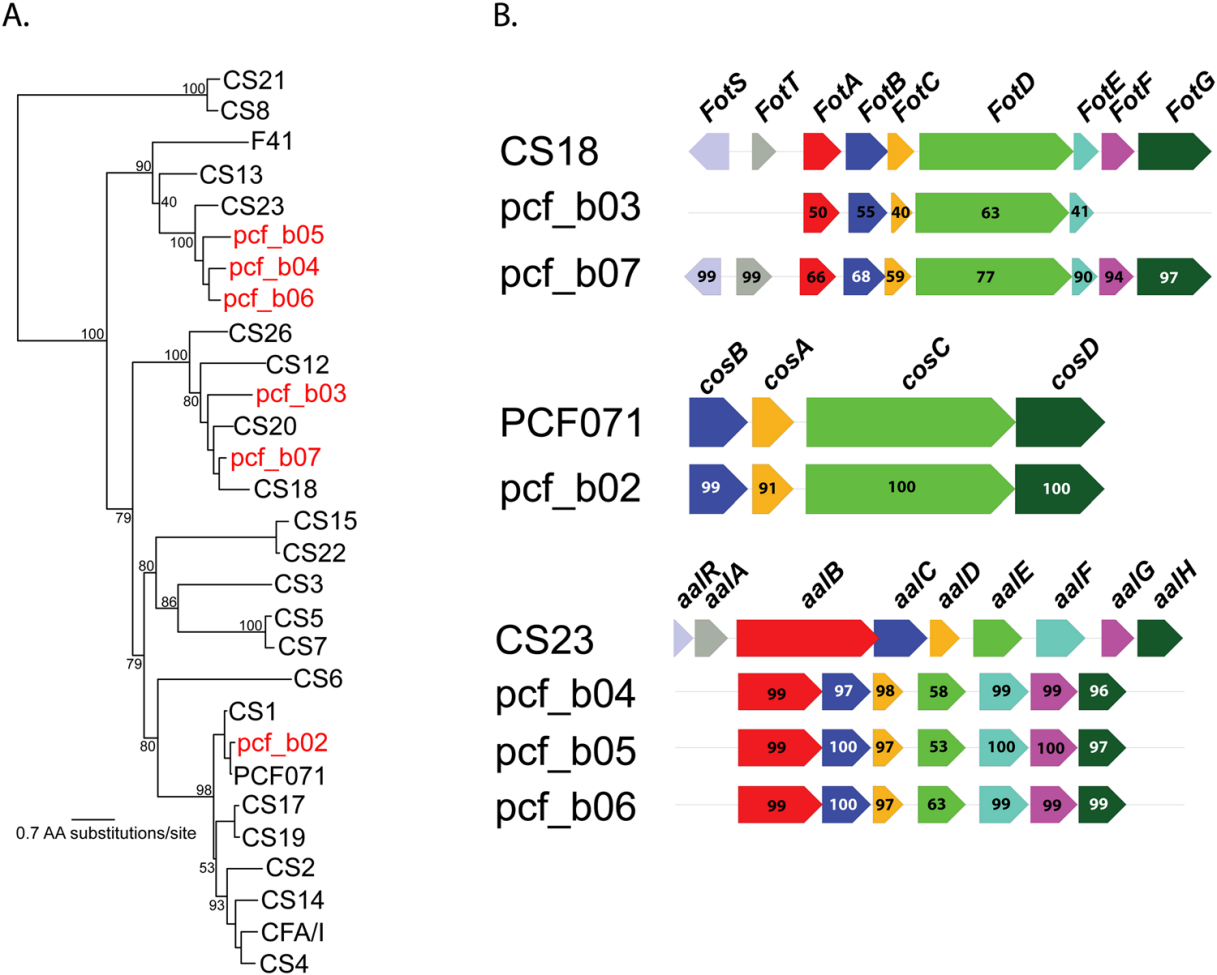
Analysis of novel putative colonization factors (CFs) identified in isolates sequenced in the current study. (**A**) A phylogenetic tree inferred from an alignment of peptide sequences from previously described CF major structural subunits, shown in black, and sequences from new putative CFs, shown in red. Sequences were aligned with MUSCLE^72^ and a phylogeny was inferred with RAxML^60^ with 100 bootstrap replicates. (**B**) Structural organization of novel putative CFs. Reference CFs were used to organize novel putative CFs. Numbers indicate the percent BLAST identity of protein sequences. The structure of novel putative CFs were identified from Prokka^71^ annotation.

Functional screening for common CF types (Table S3, Fig. 2) identified a significant proportion of the isolates (n = 21 of 94) sequenced in this study did not encode a recognizable CF (peptide BSR value < 0.90). However, several sequences from these isolates did share homology (peptide BSR > 0.50) with the structural subunits from known CFs (Table S3)^9^. Further analysis of contigs from genomes encoding potential CF structural subunits identified extended homology to previously characterized CF gene clusters. Phylogenetic (Fig. 4A) and direct amino acid comparisons to previously annotated CFs identified six new putative colonization factors (Table 3, Fig. 4B). Based on divergence in the peptide sequences of structural subunits these putative colonization factors were named pcf (putative colonization factor), b (Bangladesh), and a number [1–6]. When the complete CF gene cluster structure of each new CF was examined, a similar gene order and cluster structure was observed (Fig. 4B). The contigs in the draft assembly that contained these gene clusters often only contained the CF cluster structure; in the case of pcf_b03, pcf_b04, and pcf_b05, homologs were seen for *aalR* and *aalA* (part of the CS23 CF operon), but were present on different contigs. This suggests that these CF-containing regions are flanked by regions that were not resolved during the genome assembly, and are possibly repetitive elements or insertion sequences, as has been highlighted as a common genomic feature in previous studies^11, 29^. Of the six novel CF clusters, three putative CF clusters showed limited homology ( < 50% AA identity) to the CS23 CF^40^.

**Table 3:** Six Novel Colonization Factor Gene Clusters Identified and Prevalence in Isolates.

name	accession	positive genomes
pcf_b01	WP_001493678	P0302293_3, P0302308_2
pcf_b02	WP_001701908	BCE001_MS16, BCE002_MS12
pcf_b03	WP_001377911	178850, C_34666, P0299917_2, 2864350, P0299917_1, P02997067_6
pcf_b04	WP_004026086	2780750, 174900, MP020980_1, MP020980_2, MP021561_2
pcf_b05	EMW44189	2785200, 2788150
pcf_b06	WP_001741098	180050

A LS-BSR screen of novel putative CFs against a collection (n = 223) of confirmed ETEC genomes (this study and^31^, demonstrated that pcf_b02 was conserved (peptide BSR value > 0.95) in only four genomes and pcf_b06 was conserved in only two genomes (Supplementary Data File 4); all other novel CFs were absent from this genomic dataset. This demonstrates that some of the putative CFs are broadly distributed temporally and geographically, while the other CFs may be unique or specific to Bangladesh.

When the novel putative CFs (Table 3) were combined with known CFs (Table S3), 91 of the 94 isolates sequenced in this study were assigned a CF type based on a peptide BSR value >= 0.98 (Fig. 2, Table S1); however, three isolates were still classified as CF negative, based on an absence of homology to known ETEC CFs. Collectively, these findings reaffirm the critical role of CFs in mediating intestinal colonization by ETEC, while highlighting the potential complexity in targeting CF antigens in vaccines.

## Discussion

Outcomes of ETEC infection in humans range from asymptomatic colonization and mild self-limiting diarrhea to severe cholera-like disease^41, 42^. Despite the prevalence of this pathovar in the developing world, current knowledge of genomic diversity of ETEC derives from screening culture collections for a limited number of virulence markers. The majority of these markers and virulence genes were identified or characterized in a single prototype ETEC isolate, H10407. However, this isolate appears to contain a gene repertoire that is not entirely representative of the majority of ETEC isolates surveyed^16, 28, 31^. In the current study, a diverse set of ETEC isolates were obtained and sequenced from individuals primarily from Bangladesh. These genomes were then compared in a reference-independent approach to understand the distribution of virulence and colonization factors. With the development of massively parallel genome sequencing, genomic comparisons are no longer reliant on comparisons to limited numbers of prototype reference isolates.

ETEC genomic analyses to date have almost exclusively focused on pathogenic isolates^11, 16^, although asymptomatic isolates have been described extensively in the literature^31, 43–45^. In the current study, the sequenced isolates include 84 ETEC isolates from individuals with diarrhea (symptomatic ETEC) and 10 ETEC isolates from asymptomatic individuals (asymptomatic ETEC); by including public genomes^31^, the numbers increased to 262 symptomatic and 23 asymptomatic genomes. Although the relatively low number of asymptomatic ETEC isolates precludes large-scale investigations into genes associated with disease, this study presents a framework of how to analyze large multidimensional datasets to identify genomic features positively associated with a given phenotype, such as disease presentation. These studies highlight the utility of moving beyond the single gene approach and taking a more systems biology approach to the study of pathogenesis.

The majority of the genomes generated in this study were isolated from Bangladesh and are associated with the clinical presentation at the time of isolation. Whole genome analyses attempted to identify genomic features that are conserved in isolates from individuals with diarrhea and absent in isolates from asymptomatic individuals. However, it is possible that an isolate currently identified as asymptomatic is actually a virulent isolate, to which the host has immunity, rather than a truly avirulent isolate. Multiple host factors, including nutrition and gastrointestinal microbiota composition, as well as multiple immune mediators, and blood group antigen presentation on mucosal surfaces may be as important as pathogen virulence factor content in determining clinical presentation. Collectively, host factors that impact the outcome of ETEC infections could confound identification of genomic region(s) exclusive to symptomatic ETEC isolates.

ETEC CFs have previously been associated with ETEC pathogenesis^26^ and have been the focus of intensive research and multiple vaccine trials^46^. In this study of 94 ETEC isolates, 23 CFs were identified, including six novel putative CFs. The identification of these six new CFs increases the known CF repertoire by ~25% and highlights the utility of whole genome sequencing in the identification of novel variants of important virulence factors. Additional work is needed to functionally characterize these new CFs and their potential contribution to virulence in those isolates. The sequence diversity observed, even this relatively small collection of isolates, highlights the challenges to the development of a broadly protective ETEC CF-based vaccine^47^. Interestingly, three symptomatic ETEC isolates were identified that can be characterized as truly CF negative, suggesting that these may possess previously uncharacterized adhesin molecules.

The whole genome phylogeny demonstrated the breadth of phylogenetic diversity of the ETEC pathovar in Bangladesh (Fig. 1, Figure S1). ETEC is one of the most diverse pathotypes^5, 31^ and have now been identified in all *E. coli* phylogroups, with the exception of B2 (Fig. 1). Phylogroup B2 is populated mainly with extra-intestinal *E. coli* and more specifically, urinary tract *E. coli*, and as such they do contain unique genes compared to the diarrheagenic *E. coli* phylogroups^35^, but it is unclear if these unique genes confer greater incompatibility with ETEC plasmids harboring enterotoxins.

This observed phylogenetic diversity is mostly likely driven by the significant number of mobile elements including phage and plasmids within the ETEC genomes, as well as the fact that all of the pathovar specific virulence factors are encoded on plasmids^11, 29^. While a detailed analysis of the complete plasmids is not possible with this dataset due to the sequencing method and the previous studies that indicated that there the ETEC genome contains ~5% repetitive elements and insertion sequences^29, 31^. However, it must be noted that a number of reference ETEC isolates^31^ included in this study are from outbreaks that occurred many decades apart and on different continents (Table S1 and S2), and yet the phylogenetic (Fig. 1 and Figure S1) and virulence factor patterns (Fig. 2) are very similar suggesting that there may be an optimal strategy of pathogenesis in these organisms that leads to a successful pathogen that can cause significant outbreaks. One hypothesis could be that there is constant sharing of genetic material among the non-pathogenic or evolving-pathogenic isolates within a host or the environment that only expands significantly when there is the optimal assemblage of the mobile genetic factors, bacterial chromosomal factors, and the opportunity to infect a susceptible population or host.

The results of this study have identified several new putative CFs, as well as a number of genomic regions differentially present in isolates from different clinical presentations among the ETEC in Bangladesh, as well as between ETEC and non-ETEC *E. coli* isolates. Importantly, this study highlights the difficulty of directly correlating pathogen genomics with clinical outcomes. However, these studies demonstrate the feasibility of large-scale genomic epidemiology as an essential tool for molecular characterization of these globally important pathogens.

## Materials and Methods

### Strain selection

A total of 94 isolates, 84 associated with moderate to severe diarrheal illness and 10 from asymptomatic colonization were analyzed in this study. The majority of isolates (n = 89) were collected at the International Centre for Diarrhoeal Disease Research (http://www.icddrb.org) in Dhaka, Bangladesh in between 2002 and 2011. Effort was taken to minimize the number of passages of these cultures to prevent plasmid loss or the loss of any other unstable genomic feature. These isolates provide insight into the circulating isolates in Bangladesh between 2002 and 2011. Four additional isolates, Envira 8/11, Envira 10/1, Juruá 18/2, and Juruá 20/10, obtained from outbreaks of severe, cholera-like ETEC diarrhea in the Amazon, were kindly provided by Ana C.P. Vicente^48^, and a single strain (ThroopD) from the USA, isolated from a patient with severe diarrhea^49^ was obtained courtesy of Richard Finkelstein. These five isolates represent reference ETEC isolates that have been characterized previously in the literature. Disease severity associated with individual isolates was assigned based on the clinical presentation at the time of isolation. ETEC isolates were confirmed from lactose-fermenting colonies based on assays for heat labile and heat-stable toxin genes, as described previously^50^. For some of these specimens, multiple isolates were sequenced per patient^51^; however, for this analysis, only a single non-redundant representative is examined to remove redundancy in the current dataset.

Genomes from a study on ETEC diversity by von Mentzer *et al*.^31^ were downloaded from the Sequence Read Archive (Accession: ERP000733). Reads were mapped to the three ETEC enterotoxins (Supplemental Table 3) with BWA-MEM^52^ and the per base depth of coverage was calculated with the GenomeCoverageBed method in BEDTOOLS^53^. The breadth of coverage, or percentage of each target that was covered by a minimum number of reads, was then calculated. Isolates having appropriate coverage on one of the toxin genes were considered confirmed ETEC genomes. The breadth of coverage analysis demonstrated that only 309 of these genomes contained one of the three ETEC enterotoxins at a minimum breadth of 80% (2x minimum depth of coverage). The genomes were subsequently assembled with SPAdes v3.6.0^54^. Genomes with an anomalous assembly size (<4.5 mb) or a large number of contigs (>500) were removed from the analysis. Finally, a core genome phylogeny was inferred with all remaining genomes, including genomes sequenced in this study and external references (Figure S1). Genomes with a clade designation that differed from our results were removed (Figure S1). Following this filtering pipeline, only 223 of the original 362 (61.6%) genomes from the study by von Mentzer *et al*.^31^ were used in downstream comparative genomic analyses (Table S1), including genomes associated with symptomatic (n = 178) and asymptomatic (n = 34) disease presentations.

### DNA extraction, sequencing, assembly

Genomic DNA was extracted with standard methods^55^ and sequenced on the Illumina HiSeq 2000 platform at the University of Maryland School of Medicine, Institute for Genome Sciences, Genome Resource Center using established SOPs. The resulting 100 bp reads were assembled with the Celera Assembler^56^; the resulting assemblies and corresponding accession numbers are shown in Table S1. *In silico* multi-locus sequence type (MLST) profiles were assigned with a public script (https://github.com/Victorian-Bioinformatics-Consortium/mlst) against the pubMLST *E. coli* database^57^. As the Celera Assembler will remove contigs with an anomalous coverage, all genomes were also assembled with SPAdes v3.6.

### Core genome single nucleotide polymorphism (SNP) phylogeny

SNPs were identified from all genome assemblies compared to K-12 W3110^58^. Each query genome assembly was aligned to the reference with NUCmer^59^ and a direct mapping of query to reference was constructed. A self-alignment was conducted for the reference with NUCmer and any SNPs falling within duplicated regions were filtered from subsequent analyses. All identified SNPs (n = 220,679) in all genomes (Supplementary Data File 1) compared to the reference were concatenated. A phylogeny was inferred on this concatenated SNP alignment with RAxML v8^60^ using the ASC_GTRGAMMA substitution model (Lewis correction^61^). The NASP pipeline that wraps all SNP identification methods is publically available (http://github.com/TGenNorth/NASP)^62^. The Retention Index^63^, which calculates the consistency of SNPs to a tree topology, was calculated with Phangorn^64^.

### Identification of an ETEC genomic core

Previously we reported the presence of an ETEC pathovar-specific genomic core^16^ using a reference-based approach. The current study utilizes a greater number of genomes with a reference-independent approach to identify genes differentially present in ETEC genomes. As outlined above, the 94 isolates from this study, as well as additional ETEC genomes from von Mentzer *et al*.^31^ were downloaded from public databases, assembled with SPAdes v3.6.0^54^, checked for the presence of one or more ETEC enterotoxins, and the resulting phylogenetic tree was inferred with FastTree2^65^. ETEC genomes were identified as belonging to phylogroups A or B1 based on location in the phylogenetic tree and previously typed historical isolates; corresponding non-ETEC genomes from these same phylogenetic groups were identified from PATRIC^66^. For phylogenetic clades A and B1, 253 ETEC genomes and 253 reference genomes (Table S2) were tested for common genes using a LS-BSR approach^35^. Genomic regions with a differential distribution in these phylogroups were identified using an R^67^ script (https://gist.github.com/jasonsahl/569e502a66dcab5c643f).

### Identification of ETEC genes associated with clinical presentation

ETEC genomes and reference genomes (Table S1) were tested for gene presence and absence using the LS-BSR approach^35^. Genomic regions with a differential distribution based on the observed clinical presentation were identified from the outlier R script, with p-values using a Kruskal-Wallis test (https://gist.github.com/jasonsahl/6dd3939d3bb8c83f74f5ec5eac665280#file-kruskal_wallis_v1-py). A False Detection Rate (FDR) adjusted p-value < 0.05 was considered significant.

### Identification of phylogenomic specific regions

In addition to a global comparison of all asymptomatic ETEC and symptomatic ETEC, a comparison was also made on selected individual lineages from the whole genome phylogeny that contain a combination of isolates from each clinical presentation (Table S1). Genomes in specific lineages were interrogated with the LS-BSR approach. Genomic regions with a differential distribution based on the observed clinical presentation were identified and the statistical test used was the Kruskal-Wallis test. Any FDR corrected p-value < 0.05 was considered significant.

### Colonization factor identification

A set of 21 common/previously identified ETEC colonization factors (Table S3) was collated and compared against the 136 genomes in the phylogeny (Fig. 1) with TBLASTN^68^ in order to identify the global CF profile. The BSR value^69^ was identified for each CF in each genome then visualized with the integrated tree of life (iTOL)^70^; regions with a BSR value of >0.9 were considered to be present. Contigs from draft assemblies that showed more remote homology (<90% peptide ID) to known CFs were annotated with Prokka^71^ to identify the operon structure of the homologous region.

Structural subunits from novel putative CFs were identified from the predicted coding sequences and annotation of each genome. Peptide sequences associated with previously identified CF structural subunits (Table S3) were aligned with new putative subunits identified in this study using MUSCLE^72^, and a phylogenetic tree was inferred with RAxML using the BLOSUM62 substitution matrix and 100 bootstrap replicates.

### *in silico* virulence factor screen of ETEC genomes

All verified and predicted ETEC virulence factors were compiled for interrogation of the genomes generated in this study (Table S3). The peptide sequence for each factor was aligned against all sequenced genomes with TBLASTN in conjunction with LS-BSR. The BSR value was calculated, and genes with a BSR ≥ 0.90 were considered to be highly conserved. The proportion of genes that were determined to be highly conserved in each group (clinical presentation, or phylogenomic group etc) was compared with a two-tailed significance test and the *p*-value was calculated from the resulting z-score.

### Accession numbers

The genome sequences generated in this study are deposited in GenBank under the accession numbers listed in Table S1.

## Electronic supplementary material

Supplemetal Information

Supplemental Datasets
