# Supplementary material for: Insights into enterotoxigenic Escherichia coli diversity in Bangladesh utilizing genomic epidemiology
Source: Sci Rep. 2017 Jun 13;7:3402. doi: 10.1038/s41598-017-03631-x (PMC5469772; doi:10.1038/s41598-017-03631-x)
Supplement: Supplementary file 1 — Supplemetal Information [file 41598_2017_3631_MOESM1_ESM.pdf]

**Supplemental Information for:**

# Insights into enterotoxigenic *Escherichia coli* diversity in Bangladesh utilizing genomic epidemiology

Jason W. Sahl [1,2,Ψ], Jeticia R. Sistrunk[1,Ψ], Nabilah Ibnat Baby[3], Yasmin Begum[3], Qingwei Luo[4], Alaullah Sheikh[3,5], Firdausi Qadri[3], James M. Fleckenstein[4,5,6], David A. Rasko[1*]

[1]Institute for Genome Sciences, Department of Microbiology and Immunology, University of Maryland School of Medicine, 801 W. Baltimore Street, Suite 600, Baltimore, MD, 21201; [2]Translational Genomics Research Institute, Flagstaff, AZ, 86001; [3]Centre for Vaccine Sciences, Immunology Laboratory, International Centre Center for Diarrhoeal Disease Research, Mohakhali, Dhaka 1212, Bangladesh; [4]Department of Medicine, Division of Infectious Diseases and [5]The Molecular Microbiology and Microbial Pathogenesis Program, Division of Biology and Biomedical Sciences, Washington University in St. Louis; [6]Medicine Service, Veterans Affairs Medical Center, St. Louis, MO.

Assembled sequencing data generated in this study has been deposited in GenBank under the accession numbers listed in Supplementary Table 1. Additional genomes used in the comparisons in this study have been included in Supplemental Table 2.

**Five additional datasets are included with this submission:**

**Supplemental Datafile 1**. SNP matrix output from the Northern Arizona SNP Pipeline (NASP). These SNPs were used to infer a core genome SNP phylogeny (Figure 1).

**Supplemental Datafile 2**. BSR Matrix of the 359 ETEC genomes and *E. coli* and *Shigella* references. This dataset provides some insight into which features are ETEC specific and the accessory genome associated with each isolate.

**Supplemental Datafile 3.** FASTA file of the coding region sequences used in the comparison of the 359 ETEC and reference *E. coli* and *Shigella* isolates.

**Supplemental Datafile 4**. FASTA file of the genes that distinguish symptomatic ETEC from asymptomatic ETEC in the group specific comparison as outlined in Figure 1.

**Supplemental Datafile 5**. BSR matrix of the CF gene clusters in a set (n=223) of ETEC from von Mezter paper. Data is normalized between 0 and 1 and provides an overview of the presence and absence of each CF gene cluster in this additional genomic dataset.

**There are 2 Supplemental Figures for this study.**

**Supplemental Figure S1.** A core genome phylogeny of the isolates generated in this study with a collection of external ETEC isolates.

**Supplemental Figure S2.** Phylogenomic relationships of the isolates in specific lineages and associated virulence factors as a heatmap.

**There are 4 Supplemental tables for this study.**

**Supplemental Table S1.** Accession numbers of genomes included in this study.

**Supplemental Table S2.** Accession numbers of genomes examined in this study.

**Supplemental Table S3.** Accession numbers of Colonization factors and Virulence factors screened in this study.

**Supplementary Table S4**. Annotation of regions differentially conserved between different groups of genomes. Bold entries indicate a p value < 0.05

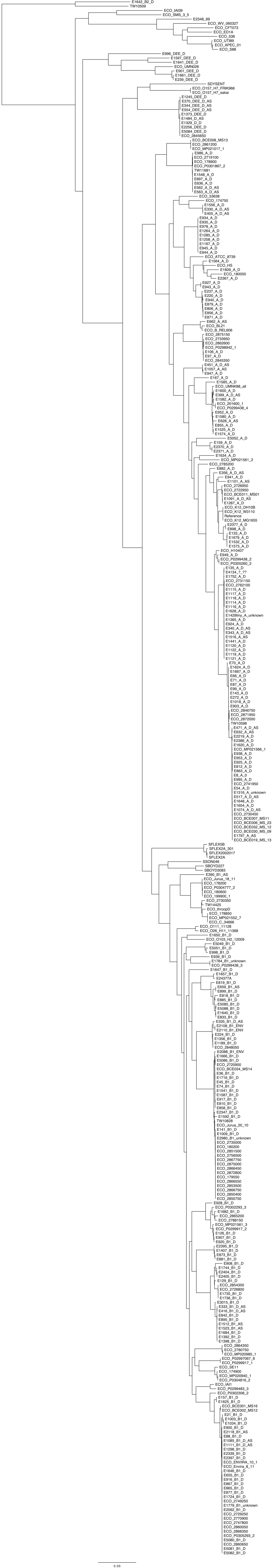

**Supplemental Figure S1.** A core genome phylogeny of genomes sequenced in this study, including a large set of external ETEC genomes. SNPs were identified in all genomes against K-12 W3110 using NASP (5). A phylogeny was then inferred on the concatenated SNPs with FastTree 2 (3).

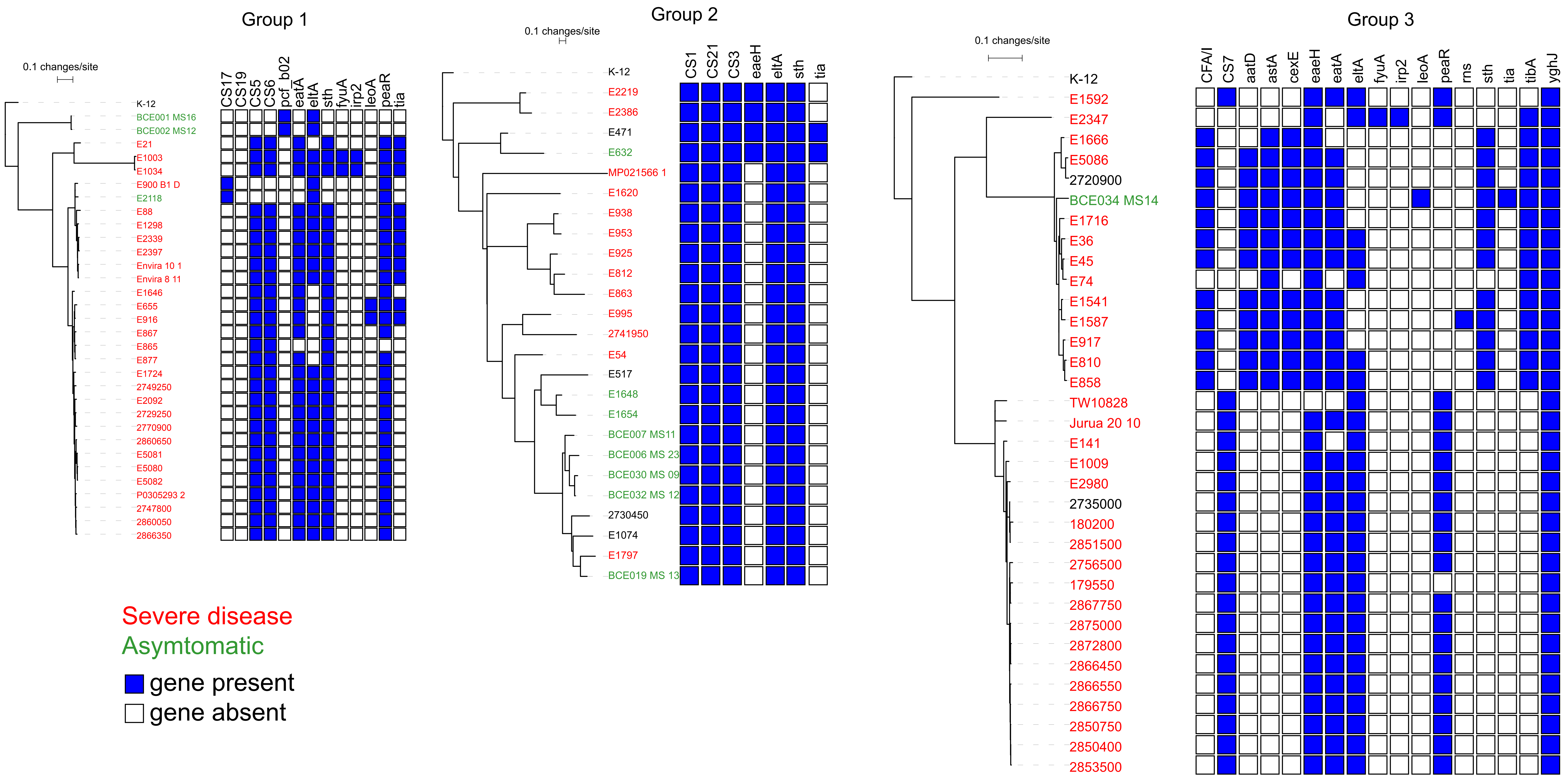

Group 1
0.1 changes/site
CS17 CS19 CS5 CS6 pcf_b02 eatA eltA sth fyuA irp2 leoA peaR tia
K-12
BCE001 MS16
BCE002 MS12
E21
E1003
E1034
E900 B1 D
E2118
E88
E1298
E2339
E2397
Envira 10 1
Envira 8 11
E1646
E655
E916
E867
E865
E877
E1724
2749250
E2092
2729250
2770900
2860650
E5081
E5080
E5082
P0305293 2
2747800
2860050
2866350
Group 2
0.1 changes/site
CS1 CS21 CS3 eaeH eltA sth tia
K-12
E2219
E2386
E471
E632
MP021566 1
E1620
E938
E953
E925
E812
E863
E995
2741950
E54
E517
E1648
E1654
BCE007 MS11
BCE006 MS 23
BCE030 MS 09
BCE032 MS 12
2730450
E1074
E1797
BCE019 MS 13
Group 3
0.1 changes/site
CFA/I CS7 aatD astA cexE eaeH eatA eltA fyuA irp2 leoA peaR rns sth tia tibA yghJ
K-12
E1592
E2347
E1666
E5086
2720900
BCE034 MS14
E1716
E36
E45
E74
E1541
E1587
E917
E810
E858
TW10828
Jurua 20 10
E141
E1009
E2980
2735000
180200
2851500
2756500
179550
2867750
2875000
2872800
2866450
2866550
2866750
2850750
2850400
2853500
Severe disease
Asymtomatic
gene present
gene absent

**Supplemental Figure S2.** Phylogenetic trees for specific phylogenomic groups with corresponding heatmaps**.** SNPs were identified in each group against K-12 W3110 using NASP. A phylogeny was then inferred on the concatenated SNPs with FastTree 2 (3)**.** The distribution of known virulence factors (Supplemental Table 2) was identified with TBLASTN (1) in conjunction with LS-BSR (4). The phylogeny was associated with the heatmap with iTOL (2).

**Supplemental References**


1. **Altschul, S. F., T. L. Madden, A. A. Schaffer, J. Zhang, Z. Zhang, W. Miller, and D. J. Lipman.** 1997. Gapped BLAST and PSI-BLAST: a new generation of protein database search programs. Nucleic Acids Res **25:**3389-402.
2. **Letunic, I., and P. Bork.** 2007. Interactive Tree Of Life (iTOL): an online tool for phylogenetic tree display and annotation. Bioinformatics **23:**127-8.
3. **Price, M. N., P. S. Dehal, and A. P. Arkin.** 2010. FastTree 2--approximately maximum-likelihood trees for large alignments. PLoS One **5:**e9490.
4. **Sahl, J. W., J. G. Caporaso, D. A. Rasko, and P. Keim.** 2014. The large-scale blast score ratio (LS-BSR) pipeline: a method to rapidly compare genetic content between bacterial genomes. PeerJ **2:e332**.
5. **Sahl, J. W., D. Lemmer, J. Travis, J. Schupp, J. Gillece, M. Aziz, E. Driebe, K. Drees, N. D. Hicks, C. Williamson, C. Hepp, D. E. Smith, C. Roe, D. M. Engelthaler, D. M. Wagner, and P. Keim.** 2016. The Northern Arizona SNP Pipeline (NASP): accurate, flexible, and rapid identification of SNPs in WGS datasets. bioRxiv.

**Supplemental Table 1. Accession numbers of genomes included in this study**

| Genome | genome type | pubMLST sequence type | Country of Isolation | Genbank Accession | SRA accession | CF profile | Clinical Group |
|---|---|---|---|---|---|---|---|
| 174750 | ETEC sequenced in current study | 1408 | Bangladesh | AQEI00000000 | SRR488683 | None | symptomatic |
| 178200 | ETEC sequenced in current study | 423 | Bangladesh | APWY00000000 | SRR446286 | CS17 | symptomatic |
| 178850 | ETEC sequenced in current study | 2851 | Bangladesh | APWZ00000000 | SRR446714 | pcf_b04 | symptomatic |
| 178900 | ETEC sequenced in current study | 2332 | Bangladesh | APXA00000000 | SRR446708 | CS21, CFA/I | symptomatic |
| 179550 | ETEC sequenced in current study | untypable | Bangladesh | APXB00000000 | SRR446711 | CS7 | symptomatic |
| 180200 | ETEC sequenced in current study | untypable | Bangladesh | APXC00000000 | SRR446715 | CS7 | symptomatic |
| 180600 | ETEC sequenced in current study | 423 | Bangladesh | AQEG00000000 | SRR446717 | CS17 | symptomatic |
| 199900_1 | ETEC sequenced in current study | 423 | Bangladesh | AQEF00000000 | SRR446718 | CS17 | symptomatic |
| 201600_1 | ETEC sequenced in current study | 100 | Bangladesh | AQFH00000000 | SRR446719 | CS14 | symptomatic |
| 2719100 | ETEC sequenced in current study | 2332 | Bangladesh | AQFG00000000 | SRR446709 | CS21, CFA/I | symptomatic |
| 2720900 | ETEC sequenced in current study | 173 | Bangladesh | AQFF00000000 | SRR446713 | CFA/I | symptomatic |
| 2722950 | ETEC sequenced in current study | 10 | Bangladesh | AQEE00000000 | SRR446721 | CFA/I | symptomatic |
| 2726950 | ETEC sequenced in current study | 10 | Bangladesh | AQED00000000 | SRR446725 | CS21, CFA/I | symptomatic |
| 2729250 | ETEC sequenced in current study | 443 | Bangladesh | AQEC00000000 | SRR446724 | CS5,CS6 | symptomatic |
| 2730350 | ETEC sequenced in current study | 23 | Bangladesh | APXD00000000 | SRR446753 | CS3,CS21 | symptomatic |
| 2730450 | ETEC sequenced in current study | 2353 | Bangladesh | APXE00000000 | SRR446751 | CS1,CS3,CS21 | symptomatic |
| 2731150 | ETEC sequenced in current study | 3931 | Bangladesh | AQEB00000000 | SRR446754 | CS6,CS8 | symptomatic |
| 2733950 | ETEC sequenced in current study | 96 | Bangladesh | APZK00000000 | SRR488358 | CS4, CS6 | symptomatic |
| 2735000 | ETEC sequenced in current study | untypable | Bangladesh | AQGH00000000 | SRR446743 | CS7 | symptomatic |
| 2741950 | ETEC sequenced in current study | 2353 | Bangladesh | APXF00000000 | SRR446748 | CS1,CS3,CS21 | symptomatic |
| 2747800 | ETEC sequenced in current study | 443 | Bangladesh | AQEA00000000 | SRR446741 | CS5,CS6 | symptomatic |
| 2749250 | ETEC sequenced in current study | 443 | Bangladesh | AQDZ00000000 | SRR446755 | CS5,CS6 | symptomatic |
| 2756500 | ETEC sequenced in current study | untypable | Bangladesh | AQDY00000000 | SRR446742 | CS7 | symptomatic |
| 2762100 | ETEC sequenced in current study | 3931 | Bangladesh | AQDX00000000 | SRR446749 | CS6,CS8 | symptomatic |
| 2770900 | ETEC sequenced in current study | 443 | Bangladesh | AQDW00000000 | SRR446744 | CS5,CS6 | symptomatic |
| 2780750 | ETEC sequenced in current study | 1196 | Bangladesh | AQDV00000000 | SRR446747 | pcf_b05 | symptomatic |
| 2785200 | ETEC sequenced in current study | 684 | Bangladesh | AQDU00000000 | SRR446745 | pcf_b06 | symptomatic |
| 2845350 | ETEC sequenced in current study | 96 | Bangladesh | AQDS00000000 | SRR446746 | CS4, CS6 | symptomatic |
| 2845650 | ETEC sequenced in current study | 182 | Bangladesh | AQDR00000000 | SRR446752 | CS6 | symptomatic |
| 2846750 | ETEC sequenced in current study | 4 | Bangladesh | AQGG00000000 | SRR446758 | CS2,CS3 | symptomatic |
| 2848050 | ETEC sequenced in current study | 94 | Bangladesh | AQDQ00000000 | SRR446759 | CS6 | symptomatic |
| 2850400 | ETEC sequenced in current study | untypable | Bangladesh | AQDP00000000 | SRR446764 | CS7 | symptomatic |
| 2850750 | ETEC sequenced in current study | untypable | Bangladesh | AQDO00000000 | SRR446763 | CS7 | symptomatic |
| 2851500 | ETEC sequenced in current study | untypable | Bangladesh | AQDN00000000 | SRR446762 | CS7 | symptomatic |
| 2853500 | ETEC sequenced in current study | untypable | Bangladesh | AQDM00000000 | SRR446789 | CS7 | symptomatic |
| 2860050 | ETEC sequenced in current study | 443 | Bangladesh | AQDL00000000 | SRR446790 | CS5,CS6 | symptomatic |
| 2860650 | ETEC sequenced in current study | 443 | Bangladesh | APXG00000000 | SRR446794 | CS5,CS6 | symptomatic |
| 2861200 | ETEC sequenced in current study | 2795 | Bangladesh | AQDK00000000 | SRR446793 | CS6 | symptomatic |
| 2862600 | ETEC sequenced in current study | 96 | Bangladesh | APXH00000000 | SRR446791 | CS4 | symptomatic |
| 2864350 | ETEC sequenced in current study | 1196 | Bangladesh | APXI00000000 | SRR446792 | pcf_b04 | symptomatic |
| 2865200 | ETEC sequenced in current study | 155 | Bangladesh | AQDJ00000000 | SRR446795 | CS23 | symptomatic |
| 2866350 | ETEC sequenced in current study | 443 | Bangladesh | APXJ00000000 | SRR446796 | CS5,CS6 | symptomatic |
| 2866450 | ETEC sequenced in current study | untypable | Bangladesh | AQDI00000000 | SRR446797 | CS7 | symptomatic |
| 2866550 | ETEC sequenced in current study | untypable | Bangladesh | AQDH00000000 | SRR446798 | CS7 | symptomatic |
| 2866750 | ETEC sequenced in current study | untypable | Bangladesh | AQDG00000000 | SRR446801 | CS7 | symptomatic |
| 2867750 | ETEC sequenced in current study | untypable | Bangladesh | AQDF00000000 | SRR446803 | CS7 | symptomatic |
| 2871950 | ETEC sequenced in current study | 4 | Bangladesh | AQDE00000000 | SRR446802 | CS2,CS3 | symptomatic |
| 2872000 | ETEC sequenced in current study | 4 | Bangladesh | AQDD00000000 | SRR446804 | CS2,CS3 | symptomatic |
| 2872800 | ETEC sequenced in current study | untypable | Bangladesh | AQDC00000000 | SRR446800 | CS7 | symptomatic |
| 2875000 | ETEC sequenced in current study | untypable | Bangladesh | AQDB00000000 | SRR446806 | CS7 | symptomatic |
| 2875150 | ETEC sequenced in current study | 1201 | Bangladesh | APXK00000000 | SRR446807 | CS4 | symptomatic |
| BCE001_MS16 | ETEC sequenced in current study | untypable | Bangladesh | AQFK00000000 | SRR446813 | pcf_b03 | asymptomatic |
| BCE002_MS12 | ETEC sequenced in current study | untypable | Bangladesh | AQDA00000000 | SRR446817 | pcf_b03 | asymptomatic |
| BCE006_MS_23 | ETEC sequenced in current study | 2353 | Bangladesh | APZM00000000 | SRR488686 | CS1,CS3,CS21 | asymptomatic |
| BCE007_MS11 | ETEC sequenced in current study | 2353 | Bangladesh | AQFJ00000000 | SRR446815 | CS1,CS3,CS21 | asymptomatic |
| BCE008_MS13 | ETEC sequenced in current study | 2795 | Bangladesh | AQFI00000000 | SRR446820 | CS6 | asymptomatic |
| BCE011_MS11 | ETEC sequenced in current study | 10 | Bangladesh | APXM00000000 | SRR446827 | CFA/I | asymptomatic |
| BCE019_MS_13 | ETEC sequenced in current study | 2353 | Bangladesh | AQCZ00000000 | SRR446829 | CS1,CS3,CS21 | asymptomatic |
| BCE030_MS_09 | ETEC sequenced in current study | 2353 | Bangladesh | APXN00000000 | SRR446831 | CS1,CS3,CS21 | asymptomatic |
| BCE032_MS_12 | ETEC sequenced in current study | 2353 | Bangladesh | APXO00000000 | SRR446828 | CS1,CS3,CS21 | asymptomatic |
| BCE034_MS14 | ETEC sequenced in current study | 173 | Bangladesh | AQCY00000000 | SRR446830 | CFA/I | asymptomatic |
| ENVIRA_10_1 | ETEC sequenced in current study | 443 | Brazil | AQFD00000000 | SRR447491 | CS5,CS6 | symptomatic |
| Envira_8_11 | ETEC sequenced in current study | 443 | Brazil | AQFC00000000 | SRR446357 | CS5,CS6 | symptomatic |
| Jurua_18_11 | ETEC sequenced in current study | 423 | Brazil | AQFB00000000 | SRR446833 | CS17 | symptomatic |
| Jurua_20_10 | ETEC sequenced in current study | 173 | Brazil | AQFA00000000 | SRR446837 | CS7 | symptomatic |
| MP020940_1 | ETEC sequenced in current study | 156 | Bangladesh | AQEZ00000000 | SRR446839 | CS23 | symptomatic |
| MP020980_1 | ETEC sequenced in current study | 1196 | Bangladesh | APZN00000000 | SRR488362 | pcf_b05 | symptomatic |
| MP021017_1 | ETEC sequenced in current study | 2795 | Bangladesh | AQEX00000000 | SRR446846 | CS6 | symptomatic |
| MP021552_7 | ETEC sequenced in current study | 2851 | Bangladesh | AQCL00000000 | SRR446866 | CS23 | symptomatic |
| MP021561_2 | ETEC sequenced in current study | 542 | Bangladesh | AQEV00000000 | SRR446879 | pcf_b05 | symptomatic |
| MP021561_3 | ETEC sequenced in current study | 58 | Bangladesh | APXP00000000 | SRR446880 | pcf_b06 | symptomatic |
| MP021566_1 | ETEC sequenced in current study | 2353 | Bangladesh | AQEU00000000 | SRR446882 | CS1,CS3,CS21 | symptomatic |
| P0298942_1 | ETEC sequenced in current study | 96 | Bangladesh | AQET00000000 | SRR446883 | CS4 | symptomatic |
| P0299438_2 | ETEC sequenced in current study | untypable | Bangladesh | AQES00000000 | SRR446898 | CS14 | symptomatic |
| P0299438_3 | ETEC sequenced in current study | 3853 | Bangladesh | APYC00000000 | SRR446899 | CS6 | symptomatic |
| P0299438_4 | ETEC sequenced in current study | 100 | Bangladesh | APYD00000000 | SRR446900 | CS14 | symptomatic |
| P0299483_3 | ETEC sequenced in current study | 517 | Bangladesh | APZS00000000 | SRR488355 | None | symptomatic |
| P02997067_6 | ETEC sequenced in current study | untypable | Bangladesh | APYJ00000000 | SRR446925 | pcf_b04 | symptomatic |
| P0299917_1 | ETEC sequenced in current study | untypable | Bangladesh | AQER00000000 | SRR446929 | pcf_b04 | symptomatic |
| P0299917_2 | ETEC sequenced in current study | 58 | Bangladesh | APYL00000000 | SRR446936 | pcf_b04 | symptomatic |
| P0301867_2 | ETEC sequenced in current study | 2332 | Bangladesh | APZU00000000 | SRR488364 | CFA/I | symptomatic |
| P0302293_3 | ETEC sequenced in current study | 155 | Bangladesh | AQAB00000000 | SRR446963 | pcf_b02 | symptomatic |
| P0302308_2 | ETEC sequenced in current study | 1623 | Bangladesh | APZE00000000 | SRR446987 | pcf_b02 | symptomatic |

| | | | | | | | |
|---|---|---|---|---|---|---|---|
| P0304777_2 | ETEC sequenced in current study | 423 | Bangladesh | AQAM00000000 | SRR447007 | CS17 | symptomatic |
| P0304816_2 | ETEC sequenced in current study | 156 | Bangladesh | AQAZ00000000 | SRR447075 | CS23 | symptomatic |
| P0305260_2 | ETEC sequenced in current study | untypable | Bangladesh | APZY00000000 | SRR488367 | None | symptomatic |
| P0305293_2 | ETEC sequenced in current study | 443 | Bangladesh | AQBX00000000 | SRR447107 | CS5,CS6 | symptomatic |
| throopD | ETEC sequenced in current study | untypable | USA | AQEJ00000000 | SRR447116 | CFA/I | symptomatic |
| 2726800 | ETEC sequenced in current study | untypable | Bangladesh | AQFE00000000 | SRR446723 | CS14 | symptomatic |
| 2854350 | ETEC sequenced in current study | untypable | Bangladesh | APZL00000000 | SRR488359 | CS14 | symptomatic |
| 174900 | ETEC sequenced in current study | 156 | Bangladesh | AQGI00000000 | SRR446300 | pcf_b05 | symptomatic |
| 2788150 | ETEC sequenced in current study | 949 | Bangladesh | AQDT00000000 | SRR446750 | pcf_b06 | symptomatic |
| C_34666 | ETEC sequenced in current study | 2851 | Bangladesh | AQCX00000000 | SRR446834 | pcf_b04 | symptomatic |
| 180050 | ETEC sequenced in current study | 1421 | Bangladesh | AQEH00000000 | SRR446716 | pcf_b07 | symptomatic |
| E2348/69 | non-ETEC | 15 | N/A | NC_011601 | N/A | N/A | N/A |
| 53638 | non-ETEC | 6 | N/A | NZ_AAKB00000000 | N/A | N/A | N/A |
| 536 | non-ETEC | 127 | N/A | NC_008253 | N/A | N/A | N/A |
| APEC_01 | non-ETEC | 95 | N/A | NC_008563 | N/A | N/A | N/A |
| ATCC_8739 | non-ETEC | 1120 | N/A | NC_010468 | N/A | N/A | N/A |
| BL21 | non-ETEC | 93 | N/A | NC_012947 | N/A | N/A | N/A |
| B_REL606 | non-ETEC | 93 | N/A | NC_012967 | N/A | N/A | N/A |
| CFT073 | non-ETEC | 73 | N/A | NC_004431 | N/A | N/A | N/A |
| ED1A | non-ETEC | 452 | N/A | NC_011745 | N/A | N/A | N/A |
| HS | non-ETEC | 46 | N/A | NC_009800 | N/A | N/A | N/A |
| IAI1 | non-ETEC | 1128 | N/A | NC_011741 | N/A | N/A | N/A |
| IAI39 | non-ETEC | 62 | N/A | NC_011750 | N/A | N/A | N/A |
| K12_DH10B | non-ETEC | 1060 | N/A | NC_010473 | N/A | N/A | N/A |
| K12_MG1655 | non-ETEC | 10 | N/A | NC_000913 | N/A | N/A | N/A |
| K12_W3110 | non-ETEC | 10 | N/A | AC_000091 | N/A | N/A | N/A |
| O103_H2_12009 | non-ETEC | 17 | N/A | NC_013353 | N/A | N/A | N/A |
| O111_11128 | non-ETEC | 16 | N/A | NC_013364 | N/A | N/A | N/A |
| O157_H7_FRIK966 | non-ETEC | 11 | N/A | NZ_ACXN00000000 | N/A | N/A | N/A |
| O157_H7_sakai | non-ETEC | 11 | N/A | NC_002695 | N/A | N/A | N/A |
| O26_H11_11368 | non-ETEC | 21 | N/A | NC_013361 | N/A | N/A | N/A |
| S88 | non-ETEC | 95 | N/A | NC_011742 | N/A | N/A | N/A |
| SE11 | non-ETEC | 156 | N/A | NC_011415 | N/A | N/A | N/A |
| SMS_3_5 | non-ETEC | 354 | N/A | NC_010498 | N/A | N/A | N/A |
| UMN026 | non-ETEC | 597 | N/A | NC_011751 | N/A | N/A | N/A |
| UTI89 | non-ETEC | 95 | N/A | NC_007946 | N/A | N/A | N/A |
| SBOYD227 | non-ETEC | 1130 | N/A | NC_007613 | N/A | N/A | N/A |
| SBOYD3083 | non-ETEC | 1129 | N/A | NC_010658 | N/A | N/A | N/A |
| SDYSENT | non-ETEC | 146 | N/A | NC_007606 | N/A | N/A | N/A |
| SFLEX2002017 | non-ETEC | 245 | N/A | CP001383 | N/A | N/A | N/A |
| SFLEX2A_301 | non-ETEC | 245 | N/A | NC_004337 | N/A | N/A | N/A |
| SFLEX2A | non-ETEC | 245 | N/A | NC_004741 | N/A | N/A | N/A |
| SFLEX5B | non-ETEC | 634 | N/A | NC_008258 | N/A | N/A | N/A |
| SSON046 | non-ETEC | 152 | N/A | NC_007384 | N/A | N/A | N/A |
| WV_060327 | non-ETEC | untypable | N/A | AERT00000000 | N/A | N/A | N/A |
| UMNK88 | reference ETEC | 100 | USA | CP002729 | N/A | K88ac | N/A |
| TW10509 | reference ETEC | 747 | India | AEHW00000000 | N/A | CS20 | N/A |
| TW10598 | reference ETEC | 4 | Guinea-Bissau | AELA00000000 | N/A | CS2,CS3,CS21 | N/A |
| TW10828 | reference ETEC | 173 | Guinea-Bissau | AELC00000000 | N/A | CS7 | N/A |
| TW11681 | reference ETEC | 2332 | Guinea-Bissau | AELD00000000 | N/A | CS21, CFA/I | N/A |
| TW14425 | reference ETEC | 23 | Guinea-Bissau | AELE000000000 | N/A | CS14 | N/A |
| E24377A | reference ETEC | 1132 | USA | NC_009801 | N/A | CS1,CS3 | N/A |
| H10407 | reference ETEC | 48 | Bangladesh | FN649414 | N/A | CFA/I | N/A |
| E99 | ETEC in Sanger study | 4 | N/A | N/A | ERS044466 | N/A | unknown |
| E998 | ETEC in Sanger study | 295 | N/A | N/A | ERS077666 | N/A | severe disease |
| E996 | ETEC in Sanger study | 3859 | N/A | N/A | ERS077665 | N/A | severe disease |
| E995 | ETEC in Sanger study | 2353 | N/A | N/A | ERS127431 | N/A | severe disease |
| E986 | ETEC in Sanger study | 2332 | N/A | N/A | ERS038956 | N/A | severe disease |
| E97 | ETEC in Sanger study | 96 | N/A | N/A | ERS044465 | N/A | severe disease |
| E978 | ETEC in Sanger study | 2368 | N/A | N/A | ERS077664 | N/A | severe disease |
| E953 | ETEC in Sanger study | 2353 | N/A | N/A | ERS038955 | N/A | severe disease |
| E952 | ETEC in Sanger study | 165 | N/A | N/A | ERS077660 | N/A | severe disease |
| E949 | ETEC in Sanger study | 48 | N/A | N/A | ERS077659 | N/A | severe disease |
| E947 | ETEC in Sanger study | 5379 | N/A | N/A | ERS077658 | N/A | severe disease |
| E945 | ETEC in Sanger study | 2368 | N/A | N/A | ERS077657 | N/A | severe disease |
| E944 | ETEC in Sanger study | 2368 | N/A | N/A | ERS077656 | N/A | severe disease |
| E943 | ETEC in Sanger study | 1497 | N/A | N/A | ERS044499 | N/A | severe disease |
| E941 | ETEC in Sanger study | 43 | N/A | N/A | ERS077655 | N/A | severe disease |
| E940 | ETEC in Sanger study | 398 | N/A | N/A | ERS038954 | N/A | severe disease |
| E939 | ETEC in Sanger study | 1325 | N/A | N/A | ERS077654 | N/A | severe disease |
| E938 | ETEC in Sanger study | 2353 | N/A | N/A | ERS038953 | N/A | severe disease |
| E935 | ETEC in Sanger study | 2368 | N/A | N/A | ERS077652 | N/A | severe disease |
| E934 | ETEC in Sanger study | 2368 | N/A | N/A | ERS077651 | N/A | severe disease |
| E928 | ETEC in Sanger study | 223 | N/A | N/A | ERS044498 | N/A | severe disease |
| E927 | ETEC in Sanger study | 1497 | N/A | N/A | ERS038952 | N/A | severe disease |
| E925 | ETEC in Sanger study | 2353 | N/A | N/A | ERS038951 | N/A | severe disease |
| E924 | ETEC in Sanger study | 1312 | N/A | N/A | ERS077649 | N/A | severe disease |
| E920 | ETEC in Sanger study | 58 | N/A | N/A | ERS044497 | N/A | severe disease |
| E917 | ETEC in Sanger study | 173 | N/A | N/A | ERS038950 | N/A | severe disease |
| E916 | ETEC in Sanger study | 443 | N/A | N/A | ERS038949 | N/A | severe disease |
| E908 | ETEC in Sanger study | 278 | N/A | N/A | ERS077648 | N/A | severe disease |
| E907 | ETEC in Sanger study | 5607 | N/A | N/A | ERS044496 | N/A | severe disease |
| E903 | ETEC in Sanger study | 4 | N/A | N/A | ERS044495 | N/A | severe disease |
| E901 | ETEC in Sanger study | 1380 | N/A | N/A | ERS044494 | N/A | severe disease |
| E900 | ETEC in Sanger study | 443 | N/A | N/A | ERS044493 | N/A | severe disease |

| | | | | | | | |
|---|---|---|---|---|---|---|---|
| E8 | ETEC in Sanger study | 2353 | N/A | N/A | ERS038927 | N/A | severe disease |
| E899 | ETEC in Sanger study | 641 | N/A | N/A | ERS077646 | N/A | severe disease |
| E898 | ETEC in Sanger study | 5264 | N/A | N/A | ERS077645 | N/A | severe disease |
| E897 | ETEC in Sanger study | 5532 | N/A | N/A | ERS038948 | N/A | severe disease |
| E895 | ETEC in Sanger study | 316 | N/A | N/A | ERS044492 | N/A | severe disease |
| E88 | ETEC in Sanger study | 443 | N/A | N/A | ERS038932 | N/A | severe disease |
| E885 | ETEC in Sanger study | 641 | N/A | N/A | ERS077638 | N/A | severe disease |
| E882 | ETEC in Sanger study | 5239 | N/A | N/A | ERS044490 | N/A | severe disease |
| E881 | ETEC in Sanger study | 940 | N/A | N/A | ERS077635 | N/A | severe disease |
| E87 | ETEC in Sanger study | 4 | N/A | N/A | ERS044464 | N/A | severe disease |
| E879 | ETEC in Sanger study | 398 | N/A | N/A | ERS038947 | N/A | severe disease |
| E877 | ETEC in Sanger study | 443 | N/A | N/A | ERS038946 | N/A | severe disease |
| E873 | ETEC in Sanger study | 940 | N/A | N/A | ERS077633 | N/A | severe disease |
| E871 | ETEC in Sanger study | 398 | N/A | N/A | ERS044489 | N/A | severe disease |
| E867 | ETEC in Sanger study | 443 | N/A | N/A | ERS044488 | N/A | severe disease |
| E865 | ETEC in Sanger study | 443 | N/A | N/A | ERS077632 | N/A | severe disease |
| E863 | ETEC in Sanger study | 2353 | N/A | N/A | ERS038945 | N/A | severe disease |
| E858 | ETEC in Sanger study | 5272 | N/A | N/A | ERS044487 | N/A | severe disease |
| E856 | ETEC in Sanger study | 398 | N/A | N/A | ERS044486 | N/A | severe disease |
| E855 | ETEC in Sanger study | 165 | N/A | N/A | ERS077630 | N/A | severe disease |
| E842 | ETEC in Sanger study | 316 | N/A | N/A | ERS044485 | N/A | severe disease |
| E833 | ETEC in Sanger study | 641 | N/A | N/A | ERS077628 | N/A | severe disease |
| E819 | ETEC in Sanger study | 0 | N/A | N/A | ERS077625 | N/A | severe disease |
| E818 | ETEC in Sanger study | 3857 | N/A | N/A | ERS077624 | N/A | severe disease |
| E812 | ETEC in Sanger study | 2353 | N/A | N/A | ERS044482 | N/A | severe disease |
| E810 | ETEC in Sanger study | 5272 | N/A | N/A | ERS044481 | N/A | severe disease |
| E806 | ETEC in Sanger study | 398 | N/A | N/A | ERS044480 | N/A | severe disease |
| E74 | ETEC in Sanger study | 173 | N/A | N/A | ERS044461 | N/A | severe disease |
| E71 | ETEC in Sanger study | 4 | N/A | N/A | ERS038931 | N/A | severe disease |
| E70 | ETEC in Sanger study | 4 | N/A | N/A | ERS038930 | N/A | severe disease |
| E66 | ETEC in Sanger study | 4 | N/A | N/A | ERS044460 | N/A | severe disease |
| E662 | ETEC in Sanger study | 93 | N/A | N/A | ERS077618 | N/A | severe disease |
| E659 | ETEC in Sanger study | 641 | N/A | N/A | ERS077617 | N/A | asymtomatic |
| E655 | ETEC in Sanger study | 443 | N/A | N/A | ERS038944 | N/A | asymtomatic |
| E636 | ETEC in Sanger study | 2332 | N/A | N/A | ERS038943 | N/A | severe disease |
| E632 | ETEC in Sanger study | 4 | N/A | N/A | ERS038942 | N/A | severe disease |
| E628 | ETEC in Sanger study | 165 | N/A | N/A | ERS077614 | N/A | asymtomatic |
| E563 | ETEC in Sanger study | 2332 | N/A | N/A | ERS038941 | N/A | asymtomatic |
| E562 | ETEC in Sanger study | 2332 | N/A | N/A | ERS038940 | N/A | unknown |
| E554 | ETEC in Sanger study | 182 | N/A | N/A | ERS038939 | N/A | unknown |
| E54 | ETEC in Sanger study | 2353 | N/A | N/A | ERS038929 | N/A | unknown |
| E517 | ETEC in Sanger study | 2353 | N/A | N/A | ERS038938 | N/A | severe disease |
| E5088 | ETEC in Sanger study | 1490 | N/A | N/A | ERS055673 | N/A | unknown |
| E5086 | ETEC in Sanger study | 173 | N/A | N/A | ERS055671 | N/A | severe disease |
| E5085 | ETEC in Sanger study | 1490 | N/A | N/A | ERS055670 | N/A | severe disease |
| E5084 | ETEC in Sanger study | 182 | N/A | N/A | ERS055669 | N/A | severe disease |
| E5082 | ETEC in Sanger study | 443 | N/A | N/A | ERS055668 | N/A | severe disease |
| E5081 | ETEC in Sanger study | 443 | N/A | N/A | ERS055667 | N/A | severe disease |
| E5080 | ETEC in Sanger study | 443 | N/A | N/A | ERS055666 | N/A | severe disease |
| E5052 | ETEC in Sanger study | 10 | N/A | N/A | ERS055665 | N/A | severe disease |
| E5051 | ETEC in Sanger study | 295 | N/A | N/A | ERS055664 | N/A | severe disease |
| E5049 | ETEC in Sanger study | 5270 | N/A | N/A | ERS055663 | N/A | severe disease |
| E471 | ETEC in Sanger study | 4 | N/A | N/A | ERS038937 | N/A | severe disease |
| E45 | ETEC in Sanger study | 173 | N/A | N/A | ERS044459 | N/A | unknown |
| E451 | ETEC in Sanger study | 3856 | N/A | N/A | ERS077598 | N/A | severe disease |
| E416 | ETEC in Sanger study | 316 | N/A | N/A | ERS038936 | N/A | unknown |
| E4134 | ETEC in Sanger study | 1312 | N/A | N/A | ERS077774 | N/A | unknown |
| E405 | ETEC in Sanger study | 731 | N/A | N/A | ERS077594 | N/A | unknown |
| E399 | ETEC in Sanger study | 100 | N/A | N/A | ERS077593 | N/A | unknown |
| E390 | ETEC in Sanger study | 3855 | N/A | N/A | ERS077591 | N/A | unknown |
| E370 | ETEC in Sanger study | 182 | N/A | N/A | ERS038935 | N/A | asymtomatic |
| E36 | ETEC in Sanger study | 173 | N/A | N/A | ERS044458 | N/A | unknown |
| E356 | ETEC in Sanger study | 5265 | N/A | N/A | ERS077588 | N/A | unknown |
| E344 | ETEC in Sanger study | 182 | N/A | N/A | ERS038934 | N/A | unknown |
| E343 | ETEC in Sanger study | 1312 | N/A | N/A | ERS077585 | N/A | unknown |
| E340 | ETEC in Sanger study | 1312 | N/A | N/A | ERS077584 | N/A | unknown |
| E335 | ETEC in Sanger study | 94 | N/A | N/A | ERS077582 | N/A | unknown |
| E333 | ETEC in Sanger study | 316 | N/A | N/A | ERS038933 | N/A | unknown |
| E330 | ETEC in Sanger study | 731 | N/A | N/A | ERS077581 | N/A | unknown |
| E3015 | ETEC in Sanger study | 316 | N/A | N/A | ERS077773 | N/A | severe disease |
| E2980 | ETEC in Sanger study | 5305 | N/A | N/A | ERS055659 | N/A | unknown |
| E272 | ETEC in Sanger study | 4 | N/A | N/A | ERS044479 | N/A | severe disease |
| E2405 | ETEC in Sanger study | 328 | N/A | N/A | ERS077771 | N/A | severe disease |
| E2404 | ETEC in Sanger study | 328 | N/A | N/A | ERS077770 | N/A | severe disease |
| E239 | ETEC in Sanger study | 394 | N/A | N/A | ERS077578 | N/A | severe disease |
| E2397 | ETEC in Sanger study | 443 | N/A | N/A | ERS055658 | N/A | severe disease |
| E2395 | ETEC in Sanger study | 940 | N/A | N/A | ERS077769 | N/A | severe disease |
| E2386 | ETEC in Sanger study | 4 | N/A | N/A | ERS055657 | N/A | severe disease |
| E237 | ETEC in Sanger study | 398 | N/A | N/A | ERS044477 | N/A | severe disease |
| E2377 | ETEC in Sanger study | 10 | N/A | N/A | ERS077765 | N/A | severe disease |
| E2371 | ETEC in Sanger study | 218 | N/A | N/A | ERS077764 | N/A | severe disease |
| E2370 | ETEC in Sanger study | 218 | N/A | N/A | ERS163317 | N/A | severe disease |
| E2367 | ETEC in Sanger study | 46 | N/A | N/A | ERS077762 | N/A | severe disease |
| E2347 | ETEC in Sanger study | 173 | N/A | N/A | ERS055655 | N/A | severe disease |
| E2339 | ETEC in Sanger study | 443 | N/A | N/A | ERS055654 | N/A | severe disease |

| | | | | | | | |
|---|---|---|---|---|---|---|---|
| E2256 | ETEC in Sanger study | 182 | N/A | N/A | ERS055653 | N/A | severe disease |
| E224 | ETEC in Sanger study | 448 | N/A | N/A | ERS044476 | N/A | severe disease |
| E2219 | ETEC in Sanger study | 4 | N/A | N/A | ERS055652 | N/A | severe disease |
| E220 | ETEC in Sanger study | 398 | N/A | N/A | ERS044475 | N/A | severe disease |
| E21 | ETEC in Sanger study | 443 | N/A | N/A | ERS044456 | N/A | severe disease |
| E2118 | ETEC in Sanger study | 443 | N/A | N/A | ERS055649 | N/A | asymtomatic |
| E2110 | ETEC in Sanger study | 94 | N/A | N/A | ERS077759 | N/A | unknown |
| E2108 | ETEC in Sanger study | 94 | N/A | N/A | ERS077758 | N/A | unknown |
| E2092 | ETEC in Sanger study | 443 | N/A | N/A | ERS055648 | N/A | severe disease |
| E2088 | ETEC in Sanger study | 173 | N/A | N/A | ERS055647 | N/A | unknown |
| E1797 | ETEC in Sanger study | 2353 | N/A | N/A | ERS055640 | N/A | asymtomatic |
| E1784 | ETEC in Sanger study | 3853 | N/A | N/A | ERS038974 | N/A | unknown |
| E1779 | ETEC in Sanger study | 443 | N/A | N/A | ERS038973 | N/A | unknown |
| E1752 | ETEC in Sanger study | 1312 | N/A | N/A | ERS077755 | N/A | severe disease |
| E1744 | ETEC in Sanger study | 328 | N/A | N/A | ERS077754 | N/A | severe disease |
| E1736 | ETEC in Sanger study | 5396 | N/A | N/A | ERS077752 | N/A | severe disease |
| E1735 | ETEC in Sanger study | 5396 | N/A | N/A | ERS077751 | N/A | severe disease |
| E1724 | ETEC in Sanger study | 443 | N/A | N/A | ERS055635 | N/A | severe disease |
| E1716 | ETEC in Sanger study | 173 | N/A | N/A | ERS038972 | N/A | severe disease |
| E1684 | ETEC in Sanger study | 316 | N/A | N/A | ERS077748 | N/A | severe disease |
| E1682 | ETEC in Sanger study | 616 | N/A | N/A | ERS077747 | N/A | severe disease |
| E167 | ETEC in Sanger study | 5460 | N/A | N/A | ERS077743 | N/A | severe disease |
| E1679 | ETEC in Sanger study | 10 | N/A | N/A | ERS163315 | N/A | severe disease |
| E1667 | ETEC in Sanger study | 4 | N/A | N/A | ERS055634 | N/A | severe disease |
| E1666 | ETEC in Sanger study | 173 | N/A | N/A | ERS077742 | N/A | severe disease |
| E1661 | ETEC in Sanger study | 394 | N/A | N/A | ERS077741 | N/A | severe disease |
| E1657 | ETEC in Sanger study | 0 | N/A | N/A | ERS055633 | N/A | severe disease |
| E1654 | ETEC in Sanger study | 2353 | N/A | N/A | ERS044454 | N/A | severe disease |
| E1650 | ETEC in Sanger study | 3863 | N/A | N/A | ERS077739 | N/A | severe disease |
| E1648 | ETEC in Sanger study | 2353 | N/A | N/A | ERS077738 | N/A | severe disease |
| E1647 | ETEC in Sanger study | 3862 | N/A | N/A | ERS077737 | N/A | severe disease |
| E1646 | ETEC in Sanger study | 443 | N/A | N/A | ERS038971 | N/A | severe disease |
| E1642 | ETEC in Sanger study | 5594 | N/A | N/A | ERS077736 | N/A | severe disease |
| E1641 | ETEC in Sanger study | 38 | N/A | N/A | ERS077735 | N/A | severe disease |
| E1640 | ETEC in Sanger study | 641 | N/A | N/A | ERS077734 | N/A | severe disease |
| E1634 | ETEC in Sanger study | 206 | N/A | N/A | ERS077731 | N/A | severe disease |
| E1628 | ETEC in Sanger study | 1312 | N/A | N/A | ERS077730 | N/A | severe disease |
| E1625 | ETEC in Sanger study | 205 | N/A | N/A | ERS077729 | N/A | severe disease |
| E1624 | ETEC in Sanger study | 4 | N/A | N/A | ERS038970 | N/A | severe disease |
| E1620 | ETEC in Sanger study | 5348 | N/A | N/A | ERS055630 | N/A | severe disease |
| E1609 | ETEC in Sanger study | 3861 | N/A | N/A | ERS077723 | N/A | severe disease |
| E1600 | ETEC in Sanger study | 100 | N/A | N/A | ERS077720 | N/A | severe disease |
| E159 | ETEC in Sanger study | 218 | N/A | N/A | ERS077713 | N/A | severe disease |
| E1597 | ETEC in Sanger study | 5336 | N/A | N/A | ERS077717 | N/A | severe disease |
| E1592 | ETEC in Sanger study | 0 | N/A | N/A | ERS077714 | N/A | severe disease |
| E1587 | ETEC in Sanger study | 173 | N/A | N/A | ERS077712 | N/A | severe disease |
| E1585 | ETEC in Sanger study | 3860 | N/A | N/A | ERS077710 | N/A | severe disease |
| E1582 | ETEC in Sanger study | 100 | N/A | N/A | ERS077709 | N/A | severe disease |
| E1580 | ETEC in Sanger study | 165 | N/A | N/A | ERS077707 | N/A | severe disease |
| E157 | ETEC in Sanger study | 205 | N/A | N/A | ERS077703 | N/A | severe disease |
| E1574 | ETEC in Sanger study | 750 | N/A | N/A | ERS077705 | N/A | severe disease |
| E1573 | ETEC in Sanger study | 10 | N/A | N/A | ERS077704 | N/A | severe disease |
| E1564 | ETEC in Sanger study | 609 | N/A | N/A | ERS077702 | N/A | severe disease |
| E1556 | ETEC in Sanger study | 0 | N/A | N/A | ERS077700 | N/A | severe disease |
| E1548 | ETEC in Sanger study | 2332 | N/A | N/A | ERS038969 | N/A | severe disease |
| E1541 | ETEC in Sanger study | 173 | N/A | N/A | ERS038968 | N/A | severe disease |
| E1532 | ETEC in Sanger study | 10 | N/A | N/A | ERS077694 | N/A | severe disease |
| E1525 | ETEC in Sanger study | 750 | N/A | N/A | ERS077692 | N/A | severe disease |
| E1523 | ETEC in Sanger study | 316 | N/A | N/A | ERS206755 | N/A | asymtomatic |
| E1516 | ETEC in Sanger study | 1312 | N/A | N/A | ERS206754 | N/A | asymtomatic |
| E1512 | ETEC in Sanger study | 316 | N/A | N/A | ERS206753 | N/A | asymtomatic |
| E1484 | ETEC in Sanger study | 182 | N/A | N/A | ERS206752 | N/A | asymtomatic |
| E1441 | ETEC in Sanger study | 1312 | N/A | N/A | ERS206747 | N/A | severe disease |
| E143 | ETEC in Sanger study | 4 | N/A | N/A | ERS044473 | N/A | severe disease |
| E1429tiny | ETEC in Sanger study | 1312 | N/A | N/A | ERS163313 | N/A | unknown |
| E141 | ETEC in Sanger study | 5305 | N/A | N/A | ERS044472 | N/A | severe disease |
| E1407 | ETEC in Sanger study | 940 | N/A | N/A | ERS077688 | N/A | severe disease |
| E1398 | ETEC in Sanger study | 316 | N/A | N/A | ERS077687 | N/A | severe disease |
| E1392 | ETEC in Sanger study | 316 | N/A | N/A | ERS038967 | N/A | severe disease |
| E1373 | ETEC in Sanger study | 182 | N/A | N/A | ERS038966 | N/A | severe disease |
| E1365 | ETEC in Sanger study | 1312 | N/A | N/A | ERS077686 | N/A | severe disease |
| E135 | ETEC in Sanger study | 1312 | N/A | N/A | ERS044471 | N/A | severe disease |
| E1356 | ETEC in Sanger study | 94 | N/A | N/A | ERS077683 | N/A | severe disease |
| E133 | ETEC in Sanger study | 10 | N/A | N/A | ERS044470 | N/A | severe disease |
| E1329 | ETEC in Sanger study | 182 | N/A | N/A | ERS206746 | N/A | severe disease |
| E1316 | ETEC in Sanger study | 2353 | N/A | N/A | ERS127432 | N/A | unknown |
| E129 | ETEC in Sanger study | 278 | N/A | N/A | ERS044469 | N/A | severe disease |
| E1298 | ETEC in Sanger study | 443 | N/A | N/A | ERS038965 | N/A | severe disease |
| E1287 | ETEC in Sanger study | 10 | N/A | N/A | ERS038964 | N/A | severe disease |
| E1285 | ETEC in Sanger study | 2368 | N/A | N/A | ERS077680 | N/A | severe disease |
| E126 | ETEC in Sanger study | 58 | N/A | N/A | ERS044468 | N/A | severe disease |
| E1264 | ETEC in Sanger study | 2368 | N/A | N/A | ERS077677 | N/A | severe disease |
| E1258 | ETEC in Sanger study | 2368 | N/A | N/A | ERS077676 | N/A | severe disease |
| E1245 | ETEC in Sanger study | 182 | N/A | N/A | ERS038963 | N/A | severe disease |
| E1189 | ETEC in Sanger study | 94 | N/A | N/A | ERS038962 | N/A | severe disease |

| | | | | | | | |
|---|---|---|---|---|---|---|---|
| E1167 | ETEC in Sanger study | 2368 | N/A | N/A | ERS077671 | N/A | severe disease |
| E1122 | ETEC in Sanger study | 5238 | N/A | N/A | ERS206744 | N/A | severe disease |
| E1121 | ETEC in Sanger study | 5238 | N/A | N/A | ERS206743 | N/A | severe disease |
| E1120 | ETEC in Sanger study | 5238 | N/A | N/A | ERS206742 | N/A | severe disease |
| E1119 | ETEC in Sanger study | 5238 | N/A | N/A | ERS206741 | N/A | severe disease |
| E1118 | ETEC in Sanger study | 3931 | N/A | N/A | ERS206740 | N/A | severe disease |
| E1117 | ETEC in Sanger study | 3931 | N/A | N/A | ERS206739 | N/A | severe disease |
| E1116 | ETEC in Sanger study | 3931 | N/A | N/A | ERS206738 | N/A | severe disease |
| E1115 | ETEC in Sanger study | 3931 | N/A | N/A | ERS206737 | N/A | severe disease |
| E1114 | ETEC in Sanger study | 3931 | N/A | N/A | ERS206736 | N/A | severe disease |
| E1111 | ETEC in Sanger study | 443 | N/A | N/A | ERS038961 | N/A | unknown |
| E1101 | ETEC in Sanger study | 167 | N/A | N/A | ERS077669 | N/A | asymtomatic |
| E1091 | ETEC in Sanger study | 10 | N/A | N/A | ERS038960 | N/A | unknown |
| E1085 | ETEC in Sanger study | 443 | N/A | N/A | ERS038959 | N/A | unknown |
| E1074 | ETEC in Sanger study | 2353 | N/A | N/A | ERS038958 | N/A | unknown |
| E106 | ETEC in Sanger study | 96 | N/A | N/A | ERS044455 | N/A | severe disease |
| E1057 | ETEC in Sanger study | 5379 | N/A | N/A | ERS077667 | N/A | asymtomatic |
| E1034 | ETEC in Sanger study | 443 | N/A | N/A | ERS038957 | N/A | severe disease |
| E1018 | ETEC in Sanger study | 4 | N/A | N/A | ERS055619 | N/A | severe disease |
| E1009 | ETEC in Sanger study | 5305 | N/A | N/A | ERS044501 | N/A | severe disease |
| E1003 | ETEC in Sanger study | 443 | N/A | N/A | ERS044500 | N/A | severe disease |

| Supplemental Table 2. Accession numbers of genomes examined in this study. | | |
|---|---|---|
| Genome | Accession | Group |
| BCE001_MS16 | AQFK00000000 | ETEC |
| BCE002_MS12 | AQDA00000000 | ETEC |
| BCE006_MS_23 | APZM00000000 | ETEC |
| BCE007_MS11 | AQFJ00000000 | ETEC |
| BCE008_MS13 | AQFI00000000 | ETEC |
| BCE011_MS11 | APXM00000000 | ETEC |
| BCE019_MS_13 | AQCZ00000000 | ETEC |
| BCE030_MS_09 | APXN00000000 | ETEC |
| BCE032_MS_12 | APXO00000000 | ETEC |
| BCE034_MS14 | AQCY00000000 | ETEC |
| C_34666 | AQCX00000000 | ETEC |
| E24377A | NC_009801 | ETEC |
| 174750 | AQEI00000000 | ETEC |
| 174900 | AQGI00000000 | ETEC |
| 178850 | APWZ00000000 | ETEC |
| 178900 | APXA00000000 | ETEC |
| 180050 | AQEH00000000 | ETEC |
| 180600 | AQEG00000000 | ETEC |
| 199900_1 | AQEF00000000 | ETEC |
| 201600_1 | AQFH00000000 | ETEC |
| 2719100 | AQFG00000000 | ETEC |
| 2720900 | AQFF00000000 | ETEC |
| 2722950 | AQEE00000000 | ETEC |
| 2726800 | AQFE00000000 | ETEC |
| 2726950 | AQED00000000 | ETEC |
| 2729250 | AQEC00000000 | ETEC |
| 2730350 | APXD00000000 | ETEC |
| 2730450 | APXE00000000 | ETEC |
| 2731150 | AQEB00000000 | ETEC |
| 2733950 | APZK00000000 | ETEC |
| 2735000 | AQGH00000000 | ETEC |
| 2741950 | APXF00000000 | ETEC |
| 2747800 | AQEA00000000 | ETEC |
| 2749250 | AQDZ00000000 | ETEC |
| 2756500 | AQDY00000000 | ETEC |
| 2762100 | AQDX00000000 | ETEC |
| 2770900 | AQDW00000000 | ETEC |
| 2780750 | AQDV00000000 | ETEC |
| 2785200 | AQDU00000000 | ETEC |
| 2788150 | AQDT00000000 | ETEC |
| 2845350 | AQDS00000000 | ETEC |
| 2846750 | AQGG00000000 | ETEC |
| 2848050 | AQDQ00000000 | ETEC |
| 2850400 | AQDP00000000 | ETEC |
| 2850750 | AQDO00000000 | ETEC |

| | | |
|---|---|---|
| 2851500 | AQDN00000000 | ETEC |
| 2853500 | AQDM00000000 | ETEC |
| 2854350 | APZL00000000 | ETEC |
| 2860650 | APXG00000000 | ETEC |
| 2861200 | AQDK00000000 | ETEC |
| 2862600 | APXH00000000 | ETEC |
| 2864350 | APXI00000000 | ETEC |
| 2865200 | AQDJ00000000 | ETEC |
| 2866350 | APXJ00000000 | ETEC |
| 2866450 | AQDI00000000 | ETEC |
| 2866550 | AQDH00000000 | ETEC |
| 2866750 | AQDG00000000 | ETEC |
| 2867750 | AQDF00000000 | ETEC |
| 2871950 | AQDE00000000 | ETEC |
| 2872000 | AQDD00000000 | ETEC |
| 2872800 | AQDC00000000 | ETEC |
| 2875000 | AQDB00000000 | ETEC |
| 2875150 | APXK00000000 | ETEC |
| H10407 | FN649414 | ETEC |
| ENVIRA_10_1 | AQFD00000000 | ETEC |
| Envira_8_11 | AQFC00000000 | ETEC |
| ERR029449 | ERR029449 | ETEC |
| ERR029451 | ERR029449 | ETEC |
| ERR029452 | ERR029449 | ETEC |
| ERR049156 | ERR029449 | ETEC |
| ERR049157 | ERR029449 | ETEC |
| ERR049158 | ERR029449 | ETEC |
| ERR049159 | ERR029449 | ETEC |
| ERR049160 | ERR029449 | ETEC |
| ERR049161 | ERR029449 | ETEC |
| ERR049162 | ERR029449 | ETEC |
| ERR049165 | ERR029449 | ETEC |
| ERR049166 | ERR029449 | ETEC |
| ERR049167 | ERR029449 | ETEC |
| ERR049169 | ERR029449 | ETEC |
| ERR049170 | ERR029449 | ETEC |
| ERR049171 | ERR029449 | ETEC |
| ERR049172 | ERR029449 | ETEC |
| ERR049173 | ERR029449 | ETEC |
| ERR049174 | ERR029449 | ETEC |
| ERR049175 | ERR029449 | ETEC |
| ERR049176 | ERR029449 | ETEC |
| ERR049177 | ERR029449 | ETEC |
| ERR049178 | ERR029449 | ETEC |
| ERR049179 | ERR029449 | ETEC |
| ERR052881 | ERR029449 | ETEC |
| ERR052882 | ERR029449 | ETEC |
| ERR052883 | ERR029449 | ETEC |

| | | |
|---|---|---|
| ERR052884 | ERR029449 | ETEC |
| ERR052885 | ERR029449 | ETEC |
| ERR052886 | ERR029449 | ETEC |
| ERR052887 | ERR029449 | ETEC |
| ERR052888 | ERR029449 | ETEC |
| ERR052889 | ERR029449 | ETEC |
| ERR052890 | ERR029449 | ETEC |
| ERR052891 | ERR029449 | ETEC |
| ERR052892 | ERR029449 | ETEC |
| ERR052894 | ERR029449 | ETEC |
| ERR052895 | ERR029449 | ETEC |
| ERR052897 | ERR029449 | ETEC |
| ERR052898 | ERR029449 | ETEC |
| ERR052899 | ERR029449 | ETEC |
| ERR052900 | ERR029449 | ETEC |
| ERR052901 | ERR029449 | ETEC |
| ERR052902 | ERR029449 | ETEC |
| ERR052903 | ERR029449 | ETEC |
| ERR052904 | ERR029449 | ETEC |
| ERR052905 | ERR029449 | ETEC |
| ERR052906 | ERR029449 | ETEC |
| ERR052907 | ERR029449 | ETEC |
| ERR052908 | ERR029449 | ETEC |
| ERR052909 | ERR029449 | ETEC |
| ERR052910 | ERR029449 | ETEC |
| ERR052911 | ERR029449 | ETEC |
| ERR052912 | ERR029449 | ETEC |
| ERR052913 | ERR029449 | ETEC |
| ERR052914 | ERR029449 | ETEC |
| ERR052915 | ERR029449 | ETEC |
| ERR052916 | ERR029449 | ETEC |
| ERR052918 | ERR029449 | ETEC |
| ERR052919 | ERR029449 | ETEC |
| ERR052921 | ERR029449 | ETEC |
| ERR052922 | ERR029449 | ETEC |
| ERR052923 | ERR029449 | ETEC |
| ERR052924 | ERR029449 | ETEC |
| ERR052925 | ERR029449 | ETEC |
| ERR052926 | ERR029449 | ETEC |
| ERR052927 | ERR029449 | ETEC |
| ERR052928 | ERR029449 | ETEC |
| ERR054665 | ERR029449 | ETEC |
| ERR054666 | ERR029449 | ETEC |
| ERR054667 | ERR029449 | ETEC |
| ERR054669 | ERR029449 | ETEC |
| ERR054670 | ERR029449 | ETEC |
| ERR054671 | ERR029449 | ETEC |
| ERR054672 | ERR029449 | ETEC |

| | | |
|---|---|---|
| ERR054673 | ERR029449 | ETEC |
| ERR054674 | ERR029449 | ETEC |
| ERR054675 | ERR029449 | ETEC |
| ERR054676 | ERR029449 | ETEC |
| ERR054677 | ERR029449 | ETEC |
| ERR054678 | ERR029449 | ETEC |
| ERR054679 | ERR029449 | ETEC |
| ERR054680 | ERR029449 | ETEC |
| ERR054681 | ERR029449 | ETEC |
| ERR054682 | ERR029449 | ETEC |
| ERR054683 | ERR029449 | ETEC |
| ERR054684 | ERR029449 | ETEC |
| ERR054686 | ERR029449 | ETEC |
| ERR054687 | ERR029449 | ETEC |
| ERR054688 | ERR029449 | ETEC |
| ERR054690 | ERR029449 | ETEC |
| ERR054691 | ERR029449 | ETEC |
| ERR054692 | ERR029449 | ETEC |
| ERR054693 | ERR029449 | ETEC |
| ERR054694 | ERR029449 | ETEC |
| ERR054695 | ERR029449 | ETEC |
| ERR054696 | ERR029449 | ETEC |
| ERR054697 | ERR029449 | ETEC |
| ERR054698 | ERR029449 | ETEC |
| ERR054699 | ERR029449 | ETEC |
| ERR054700 | ERR029449 | ETEC |
| ERR054701 | ERR029449 | ETEC |
| ERR054703 | ERR029449 | ETEC |
| ERR054704 | ERR029449 | ETEC |
| ERR054706 | ERR029449 | ETEC |
| ERR054707 | ERR029449 | ETEC |
| ERR054708 | ERR029449 | ETEC |
| ERR054709 | ERR029449 | ETEC |
| ERR054710 | ERR029449 | ETEC |
| ERR054711 | ERR029449 | ETEC |
| ERR054712 | ERR029449 | ETEC |
| ERR084463 | ERR029449 | ETEC |
| ERR084466 | ERR029449 | ETEC |
| ERR084467 | ERR029449 | ETEC |
| ERR084468 | ERR029449 | ETEC |
| ERR084470 | ERR029449 | ETEC |
| ERR084472 | ERR029449 | ETEC |
| ERR084473 | ERR029449 | ETEC |
| ERR084474 | ERR029449 | ETEC |
| ERR084475 | ERR029449 | ETEC |
| ERR084476 | ERR029449 | ETEC |
| ERR084477 | ERR029449 | ETEC |
| ERR084478 | ERR029449 | ETEC |

| | | |
|---|---|---|
| ERR084479 | ERR029449 | ETEC |
| ERR084480 | ERR029449 | ETEC |
| ERR084481 | ERR029449 | ETEC |
| ERR084482 | ERR029449 | ETEC |
| ERR084483 | ERR029449 | ETEC |
| ERR084484 | ERR029449 | ETEC |
| ERR084486 | ERR029449 | ETEC |
| ERR084487 | ERR029449 | ETEC |
| ERR084489 | ERR029449 | ETEC |
| ERR084490 | ERR029449 | ETEC |
| ERR084491 | ERR029449 | ETEC |
| ERR089723 | ERR029449 | ETEC |
| ERR089724 | ERR029449 | ETEC |
| ERR089726 | ERR029449 | ETEC |
| ERR089727 | ERR029449 | ETEC |
| ERR089729 | ERR029449 | ETEC |
| ERR089730 | ERR029449 | ETEC |
| ERR089732 | ERR029449 | ETEC |
| ERR089733 | ERR029449 | ETEC |
| ERR089734 | ERR029449 | ETEC |
| ERR089736 | ERR029449 | ETEC |
| ERR089738 | ERR029449 | ETEC |
| ERR089739 | ERR029449 | ETEC |
| ERR089740 | ERR029449 | ETEC |
| ERR089741 | ERR029449 | ETEC |
| ERR089742 | ERR029449 | ETEC |
| ERR089743 | ERR029449 | ETEC |
| ERR089745 | ERR029449 | ETEC |
| ERR089746 | ERR029449 | ETEC |
| ERR089748 | ERR029449 | ETEC |
| ERR089749 | ERR029449 | ETEC |
| ERR114756 | ERR029449 | ETEC |
| ERR114757 | ERR029449 | ETEC |
| ERR433366 | ERR029449 | ETEC |
| Jurua_18_11 | ERR029449 | ETEC |
| MP020940_1 | ERR029449 | ETEC |
| MP020980_1 | ERR029449 | ETEC |
| MP020980_2 | ERR029449 | ETEC |
| MP021017_1 | ERR029449 | ETEC |
| MP021552_7 | ERR029449 | ETEC |
| MP021561_2 | ERR029449 | ETEC |
| MP021561_3 | ERR029449 | ETEC |
| MP021566_1 | ERR029449 | ETEC |
| P0298942_1 | ERR029449 | ETEC |
| P0299438_2 | ERR029449 | ETEC |
| P0299438_3 | ERR029449 | ETEC |
| P0299438_4 | ERR029449 | ETEC |
| P0299483_2 | ERR029449 | ETEC |

| | | |
|---|---|---|
| P0299483_3 | ERR029449 | ETEC |
| P02997067_6 | ERR029449 | ETEC |
| P0299917_1 | ERR029449 | ETEC |
| P0299917_2 | ERR029449 | ETEC |
| P0301867_2 | ERR029449 | ETEC |
| P0302293_3 | ERR029449 | ETEC |
| P0302308_2 | ERR029449 | ETEC |
| P0304777_2 | ERR029449 | ETEC |
| P0304816_2 | ERR029449 | ETEC |
| P0305260_2 | ERR029449 | ETEC |
| P0305293_2 | ERR029449 | ETEC |
| throopD | AQEJ00000000 | ETEC |
| TW10598 | AELA00000000 | ETEC |
| TW10828 | AELC00000000 | ETEC |
| TW11681 | AELD00000000 | ETEC |
| TW14425 | AELE000000000 | ETEC |
| Escherichia_coli_01288 | AMVJ01000001 | non-ETEC |
| Escherichia_coli_101-1 | NZ_AAMK02000001 | non-ETEC |
| Escherichia_coli_113303 | AXUG01000001 | non-ETEC |
| Escherichia_coli_1180 | ADUQ01000001 | non-ETEC |
| Escherichia_coli_12264 | AEZO02000001 | non-ETEC |
| Escherichia_coli_1357 | ADUR01000001 | non-ETEC |
| Escherichia_coli_1365 | AYQK01000001 | non-ETEC |
| Escherichia_coli_148 | AYJX01000001 | non-ETEC |
| Escherichia_coli_1827-70 | NZ_ADUK01000001 | non-ETEC |
| Escherichia_coli_24168 | AFAD02000001 | non-ETEC |
| Escherichia_coli_27A | AYQZ01000001 | non-ETEC |
| Escherichia_coli_320 | AYQM01000001 | non-ETEC |
| Escherichia_coli_32608 | AEZS02000001 | non-ETEC |
| Escherichia_coli_3431 | ADUM01000001 | non-ETEC |
| Escherichia_coli_3816 | AYQL01000001 | non-ETEC |
| Escherichia_coli_3827 | AYQO01000001 | non-ETEC |
| Escherichia_coli_3834 | AYQP01000001 | non-ETEC |
| Escherichia_coli_40967 | AFAA02000001 | non-ETEC |
| Escherichia_coli_50588 | AEZK02000001 | non-ETEC |
| Escherichia_coli_50959 | AEZX02000001 | non-ETEC |
| Escherichia_coli_541-1 | AJWR01000001 | non-ETEC |
| Escherichia_coli_606 | AYQV01000001 | non-ETEC |
| Escherichia_coli_75 | AJWT01000001 | non-ETEC |
| Escherichia_coli_90111 | AEZZ02000001 | non-ETEC |
| Escherichia_coli_907357 | AXUH01000001 | non-ETEC |
| Escherichia_coli_908541 | AXTQ01000001 | non-ETEC |
| Escherichia_coli_908555 | AXTR01000001 | non-ETEC |
| Escherichia_coli_908658 | AXTX01000001 | non-ETEC |
| Escherichia_coli_909945-2 | AXUK01000001 | non-ETEC |
| Escherichia_coli_930624 | AEZT02000001 | non-ETEC |
| Escherichia_coli_950941 | AEZN01000001 | non-ETEC |
| Escherichia_coli_96154 | AEZW02000001 | non-ETEC |

| | | |
|---|---|---|
| Escherichia_coli_970246 | AEZJ02000001 | non-ETEC |
| Escherichia_coli_AD30 | AMSK01000001 | non-ETEC |
| Escherichia_coli_AI27 | AJPQ01000001 | non-ETEC |
| Escherichia_coli_APEC_O78 | CP004009 | non-ETEC |
| Escherichia_coli_ATCC_8739 | NC_010468 | non-ETEC |
| Escherichia_coli_ATCC_BAA-2196 | AYOF01000001 | non-ETEC |
| Escherichia_coli_ATCC_BAA-2215 | AZCA01000001 | non-ETEC |
| Escherichia_coli_ATCC_BAA-2219 | AYRW01000001 | non-ETEC |
| Escherichia_coli_B088 | NZ_GG749125 | non-ETEC |
| Escherichia_coli_B171 | NC_002142 | non-ETEC |
| Escherichia_coli_B799 | AHWG01000001 | non-ETEC |
| Escherichia_coli_BIDMC_37 | AYHV01000001 | non-ETEC |
| Escherichia_coli_BIDMC_39 | AYHT01000001 | non-ETEC |
| Escherichia_coli_BL21DE3_Prj-20713 | CP001509 | non-ETEC |
| Escherichia_coli_BL21-GoldDE3pLysS_AG | NC_012947 | non-ETEC |
| Escherichia_coli_B_str_REL606 | NC_012967 | non-ETEC |
| Escherichia_coli_C1214_90 | AIAC01000001 | non-ETEC |
| Escherichia_coli_C155_11 | AIAG01000001 | non-ETEC |
| Escherichia_coli_C166_11 | AICF01000001 | non-ETEC |
| Escherichia_coli_C213_10 | AIAL01000001 | non-ETEC |
| Escherichia_coli_C238_91 | AIAN01000001 | non-ETEC |
| Escherichia_coli_C260_92 | AIAO01000001 | non-ETEC |
| Escherichia_coli_C283_09 | AIAQ01000001 | non-ETEC |
| Escherichia_coli_C321deltaA | CP006698 | non-ETEC |
| Escherichia_coli_C488_07 | AIBA01000001 | non-ETEC |
| Escherichia_coli_C496_10 | AIBB01000001 | non-ETEC |
| Escherichia_coli_C497_10 | AIBC01000001 | non-ETEC |
| Escherichia_coli_C527_94 | AIBD01000001 | non-ETEC |
| Escherichia_coli_C652_10 | AIBI01000001 | non-ETEC |
| Escherichia_coli_C654_09 | AIBJ01000001 | non-ETEC |
| Escherichia_coli_C717_10 | AIBK01000001 | non-ETEC |
| Escherichia_coli_C725_88 | AIBL01000001 | non-ETEC |
| Escherichia_coli_C732_98 | AIBM01000001 | non-ETEC |
| Escherichia_coli_C743_03 | AIBN01000001 | non-ETEC |
| Escherichia_coli_C792_92 | AIBR01000001 | non-ETEC |
| Escherichia_coli_C799_92 | AIBT01000001 | non-ETEC |
| Escherichia_coli_C80_08 | AIBU01000001 | non-ETEC |
| Escherichia_coli_C807_09 | AIBV01000001 | non-ETEC |
| Escherichia_coli_C900_01 | AICC01000001 | non-ETEC |
| Escherichia_coli_DEC10A | AIGP01000001 | non-ETEC |
| Escherichia_coli_DEC10B | AIGQ01000001 | non-ETEC |
| Escherichia_coli_DEC10C | AIGR01000001 | non-ETEC |
| Escherichia_coli_DEC10D | AIGS01000001 | non-ETEC |
| Escherichia_coli_DEC10F | AIGU01000001 | non-ETEC |
| Escherichia_coli_DEC11B | AIGW01000001 | non-ETEC |
| Escherichia_coli_DEC11C | AIGX01000001 | non-ETEC |
| Escherichia_coli_DEC11D | AIGY01000001 | non-ETEC |
| Escherichia_coli_DEC12A | AIHA01000001 | non-ETEC |

| | | |
|---|---|---|
| Escherichia_coli_DEC12B | AIHB01000001 | non-ETEC |
| Escherichia_coli_DEC12D | AIHD01000001 | non-ETEC |
| Escherichia_coli_DEC12E | AIHE01000001 | non-ETEC |
| Escherichia_coli_DEC13A | AIHF01000001 | non-ETEC |
| Escherichia_coli_DEC13E | AIHJ01000001 | non-ETEC |
| Escherichia_coli_DEC14A | AIHK01000001 | non-ETEC |
| Escherichia_coli_DEC14B | AIHL01000001 | non-ETEC |
| Escherichia_coli_DEC14C | AIHM01000001 | non-ETEC |
| Escherichia_coli_DEC15A | AIHO01000001 | non-ETEC |
| Escherichia_coli_DEC15C | AIHQ01000001 | non-ETEC |
| Escherichia_coli_DEC15D | AIHR01000001 | non-ETEC |
| Escherichia_coli_DEC15E | AIHS01000001 | non-ETEC |
| Escherichia_coli_DEC6B | AIFW01000001 | non-ETEC |
| Escherichia_coli_DEC6C | AIFX01000001 | non-ETEC |
| Escherichia_coli_DEC6D | AIFY01000001 | non-ETEC |
| Escherichia_coli_DEC7B | AIGB01000001 | non-ETEC |
| Escherichia_coli_DEC7D | AIGD01000001 | non-ETEC |
| Escherichia_coli_DEC8B | AIGG01000001 | non-ETEC |
| Escherichia_coli_DEC8E | AIGJ01000001 | non-ETEC |
| Escherichia_coli_DEC9A | AIGK01000001 | non-ETEC |
| Escherichia_coli_DEC9C | AIGM01000001 | non-ETEC |
| Escherichia_coli_DEC9D | AIGN01000001 | non-ETEC |
| Escherichia_coli_DH1 | CP001637 | non-ETEC |
| Escherichia_coli_E110019 | NZ_AAJW02000001 | non-ETEC |
| Escherichia_coli_E1520 | AEHT01000001 | non-ETEC |
| Escherichia_coli_E482 | AEHU01000001 | non-ETEC |
| Escherichia_coli_ECA-0157 | AHHK01000001 | non-ETEC |
| Escherichia_coli_ECC-1470 | AHHM01000001 | non-ETEC |
| Escherichia_coli_EPECa14 | ADUN01000001 | non-ETEC |
| Escherichia_coli_EPEC_C342-62 | AKNI01000001 | non-ETEC |
| Escherichia_coli_FAP2 | AYKB01000001 | non-ETEC |
| Escherichia_coli_FBP1 | AYKC01000001 | non-ETEC |
| Escherichia_coli_FCP1 | AYKE01000001 | non-ETEC |
| Escherichia_coli_G58-1 | AFDX01000001 | non-ETEC |
| Escherichia_coli_H489 | AEHX01000001 | non-ETEC |
| Escherichia_coli_H591 | NZ_ADBB01000001 | non-ETEC |
| Escherichia_coli_H730 | AHWF01000001 | non-ETEC |
| Escherichia_coli_H736 | NZ_ADAU01000001 | non-ETEC |
| Escherichia_coli_HVH_115_4-4465997 | AVWD01000001 | non-ETEC |
| Escherichia_coli_HVH_121_4-6877826 | AVWK01000001 | non-ETEC |
| Escherichia_coli_HVH_147_4-5893887 | AVXG01000001 | non-ETEC |
| Escherichia_coli_HVH_150_4-3258106 | AVXJ01000001 | non-ETEC |
| Escherichia_coli_HVH_164_4-5953081 | AVXX01000001 | non-ETEC |
| Escherichia_coli_HVH_18_4-8589585 | AVTK01000001 | non-ETEC |
| Escherichia_coli_HVH_195_3-7155360 | AVYV01000001 | non-ETEC |
| Escherichia_coli_HVH_200_4-4449924 | AVZA01000001 | non-ETEC |
| Escherichia_coli_HVH_209_4-3062651 | AVZJ01000001 | non-ETEC |
| Escherichia_coli_HVH_221_4-3136817 | AVZT01000001 | non-ETEC |

| | | |
|---|---|---|
| Escherichia_coli_HVH_36_4-5675286 | AYLQ01000001 | non-ETEC |
| Escherichia_coli_HVH_50_4-2593475 | AYHF01000001 | non-ETEC |
| Escherichia_coli_HVH_82_4-2209276 | AVVE01000001 | non-ETEC |
| Escherichia_coli_HVH_91_4-4638751 | AVVM01000001 | non-ETEC |
| Escherichia_coli_IAI1 | NC_011741 | non-ETEC |
| Escherichia_coli_IMT8073 | ASXQ01000001 | non-ETEC |
| Escherichia_coli_IS25 | CBWG010000001 | non-ETEC |
| Escherichia_coli_J53 | AICK01000001 | non-ETEC |
| Escherichia_coli_JB1-95 | AEZV02000001 | non-ETEC |
| Escherichia_coli_KO11 | CP002516 | non-ETEC |
| Escherichia_coli_KOEGE_131_358a | AWAS01000001 | non-ETEC |
| Escherichia_coli_KOEGE_33_68a | AWAE01000001 | non-ETEC |
| Escherichia_coli_KOEGE_3_4a | AVZZ01000001 | non-ETEC |
| Escherichia_coli_KOEGE_40_102a | AWAF01000001 | non-ETEC |
| Escherichia_coli_KOEGE_7_16a | AWAA01000001 | non-ETEC |
| Escherichia_coli_KTE100 | ASVF01000001 | non-ETEC |
| Escherichia_coli_KTE107 | ASVI01000001 | non-ETEC |
| Escherichia_coli_KTE10 | ANSQ01000001 | non-ETEC |
| Escherichia_coli_KTE119 | ANUZ01000001 | non-ETEC |
| Escherichia_coli_KTE120 | ANXS01000001 | non-ETEC |
| Escherichia_coli_KTE12 | ANSS01000001 | non-ETEC |
| Escherichia_coli_KTE135 | ANWH01000001 | non-ETEC |
| Escherichia_coli_KTE136 | ANWI01000001 | non-ETEC |
| Escherichia_coli_KTE138 | ANYB01000001 | non-ETEC |
| Escherichia_coli_KTE14 | ASTU01000001 | non-ETEC |
| Escherichia_coli_KTE156 | ANVC01000001 | non-ETEC |
| Escherichia_coli_KTE171 | ANVG01000001 | non-ETEC |
| Escherichia_coli_KTE197 | ANWZ01000001 | non-ETEC |
| Escherichia_coli_KTE198 | ASUK01000001 | non-ETEC |
| Escherichia_coli_KTE212 | ANTO01000001 | non-ETEC |
| Escherichia_coli_KTE222 | ASUP01000001 | non-ETEC |
| Escherichia_coli_KTE232 | ANYS01000001 | non-ETEC |
| Escherichia_coli_KTE234 | ANTX01000001 | non-ETEC |
| Escherichia_coli_KTE29 | ANVQ01000001 | non-ETEC |
| Escherichia_coli_KTE40 | ASUE01000001 | non-ETEC |
| Escherichia_coli_KTE41 | ASUF01000001 | non-ETEC |
| Escherichia_coli_KTE42 | ANVN01000001 | non-ETEC |
| Escherichia_coli_KTE48 | ANVT01000001 | non-ETEC |
| Escherichia_coli_KTE51 | ANUD01000001 | non-ETEC |
| Escherichia_coli_KTE56 | ANUG01000001 | non-ETEC |
| Escherichia_coli_KTE61 | ASUU01000001 | non-ETEC |
| Escherichia_coli_KTE64 | ASUV01000001 | non-ETEC |
| Escherichia_coli_KTE73 | ASVA01000001 | non-ETEC |
| Escherichia_coli_KTE77 | ANUQ01000001 | non-ETEC |
| Escherichia_coli_KTE81 | ANUS01000001 | non-ETEC |
| Escherichia_coli_KTE91 | ANWC01000001 | non-ETEC |
| Escherichia_coli_LT-68 | ADUP01000001 | non-ETEC |
| Escherichia_coli_LY180 | CP006584 | non-ETEC |

| | | |
|---|---|---|
| Escherichia_coli_M919 | AEVZ01000001 | non-ETEC |
| Escherichia_coli_MS_115-1 | NZ_GG771708 | non-ETEC |
| Escherichia_coli_MS_116-1 | NZ_GG773342 | non-ETEC |
| Escherichia_coli_MS_117-3 | ADTS01000001 | non-ETEC |
| Escherichia_coli_MS_182-1 | NZ_GG771869 | non-ETEC |
| Escherichia_coli_MS_187-1 | NZ_GG772477 | non-ETEC |
| Escherichia_coli_MS_196-1 | NZ_GG773880 | non-ETEC |
| Escherichia_coli_MS_78-1 | NZ_GG773215 | non-ETEC |
| Escherichia_coli_MS_85-1 | ADWQ01000001 | non-ETEC |
| Escherichia_coli_NCCP15647 | AJMB01000001 | non-ETEC |
| Escherichia_coli_NCCP15657 | AJLU01000001 | non-ETEC |
| Escherichia_coli_O103-H25_str_CVM9340 | AJVQ01000001 | non-ETEC |
| Escherichia_coli_O103-H25_str_NIPH-11060424 | AGSG01000001 | non-ETEC |
| Escherichia_coli_O103-H2_str_12009 | NC_013353 | non-ETEC |
| Escherichia_coli_O103-H2_str_CVM9450 | AJVR01000001 | non-ETEC |
| Escherichia_coli_O104-H4_str_C227-11_Broad | AFRH01000001 | non-ETEC |
| Escherichia_coli_O104-H4_str_E11210 | AHAV01000001 | non-ETEC |
| Escherichia_coli_O104-H4_str_E9211 | AHAU01000001 | non-ETEC |
| Escherichia_coli_O10-K5L-H4_str_ATCC_23506 | CAPK01000001 | non-ETEC |
| Escherichia_coli_O111-B4_str_C4893 | AHAX01000001 | non-ETEC |
| Escherichia_coli_O111-H11_str_CFSAN001630 | AMXP01000001 | non-ETEC |
| Escherichia_coli_O111-H11_str_CVM9534 | AJVS01000001 | non-ETEC |
| Escherichia_coli_O111-H11_str_CVM9545 | AJVT01000001 | non-ETEC |
| Escherichia_coli_O111-H8_str_CFSAN001632 | AMXQ01000001 | non-ETEC |
| Escherichia_coli_O111-H8_str_CVM9602 | AKAV01000001 | non-ETEC |
| Escherichia_coli_O111-H8_str_CVM9634 | AKAW01000001 | non-ETEC |
| Escherichia_coli_O111-H-_str_11128 | NC_013364 | non-ETEC |
| Escherichia_coli_O113-H21_str_CL-3 | AGTH01000001 | non-ETEC |
| Escherichia_coli_O121-H19_str_MT2 | AGTJ01000001 | non-ETEC |
| Escherichia_coli_O127-B8_str_C4390 | AHAW01000001 | non-ETEC |
| Escherichia_coli_O157-H43_str_T22 | AHZD01000001 | non-ETEC |
| Escherichia_coli_O157_str_NCCP15738 | ASHB01000001 | non-ETEC |
| Escherichia_coli_O26-H11_str_CFSAN001629 | AMXO01000001 | non-ETEC |
| Escherichia_coli_O26-H11_str_CVM10030 | AKBA01000001 | non-ETEC |
| Escherichia_coli_O26-H11_str_CVM10224 | AKBB01000001 | non-ETEC |
| Escherichia_coli_O26-H11_str_CVM9942 | AJVW01000001 | non-ETEC |
| Escherichia_coli_O26-H11_str_CVM9952 | AKBC01000001 | non-ETEC |
| Escherichia_coli_O32-H37_str_P4 | AJQW01000001 | non-ETEC |
| Escherichia_coli_O5-K4L-H4_str_ATCC_23502 | CAPL01000001 | non-ETEC |
| Escherichia_coli_O91_[PRJNA206275] | AOUQ01000001 | non-ETEC |
| Escherichia_coli_OK1114 | AICG01000001 | non-ETEC |
| Escherichia_coli_P4-NR | AHHP01000001 | non-ETEC |
| Escherichia_coli_SCD1 | ATJZ01000001 | non-ETEC |
| Escherichia_coli_STEC_DG131-3 | AFDV01000001 | non-ETEC |
| Escherichia_coli_STEC_EH250 | AFDW01000001 | non-ETEC |
| Escherichia_coli_STEC_H18 | AFDY01000001 | non-ETEC |
| Escherichia_coli_STEC_S1191 | AFEA01000001 | non-ETEC |
| Escherichia_coli_str_K-12_substr_DH10B | NC_010473 | non-ETEC |

| | | |
|---|---|---|
| Escherichia_coli_str_K-12_substr_MC4100 | HG738867 | non-ETEC |
| Escherichia_coli_str_K-12_substr_MG1655 | NC_000913 | non-ETEC |
| Escherichia_coli_str_K-12_substr_W3110 | AC_000091 | non-ETEC |
| Escherichia_coli_TA007 | AEHY01000001 | non-ETEC |
| Escherichia_coli_TA271 | NZ_ADAZ01000001 | non-ETEC |
| Escherichia_coli_TY-2482 | AFVR01000001 | non-ETEC |
| Escherichia_coli_UMEA_3052-1 | AWAX01000001 | non-ETEC |
| Escherichia_coli_UMEA_3148-1 | AWBM01000001 | non-ETEC |
| Escherichia_coli_UMEA_3150-1 | AWBN01000001 | non-ETEC |
| Escherichia_coli_UMEA_3180-1 | AWCC01000001 | non-ETEC |
| Escherichia_coli_UMEA_3199-1 | AWCG01000001 | non-ETEC |
| Escherichia_coli_UMEA_3201-1 | AWCI01000001 | non-ETEC |
| Escherichia_coli_UMEA_3212-1 | AWCM01000001 | non-ETEC |
| Escherichia_coli_UMEA_3240-1 | AWCV01000001 | non-ETEC |
| Escherichia_coli_UMEA_3271-1 | AWDA01000001 | non-ETEC |
| Escherichia_coli_UMEA_3292-1 | AWDB01000001 | non-ETEC |
| Escherichia_coli_UMEA_3314-1 | AWDE01000001 | non-ETEC |
| Escherichia_coli_UMEA_3317-1 | AWDF01000001 | non-ETEC |
| Escherichia_coli_UMEA_3329-1 | AWDH01000001 | non-ETEC |
| Escherichia_coli_UMEA_3336-1 | AYHH01000001 | non-ETEC |
| Escherichia_coli_UMEA_3355-1 | AWDK01000001 | non-ETEC |
| Escherichia_coli_UMEA_3592-1 | AWDO01000001 | non-ETEC |
| Escherichia_coli_UMEA_3682-1 | AWDW01000001 | non-ETEC |
| Escherichia_coli_UMEA_3805-1 | AWEE01000001 | non-ETEC |
| Escherichia_coli_UMEA_3889-1 | AWEH01000001 | non-ETEC |
| Escherichia_coli_W | CP002185 | non-ETEC |
| Escherichia_coli_W_Prj-42709 | NZ_AEDF01000001 | non-ETEC |
| Escherichia_coli_XH001 | AFYG01000001 | non-ETEC |
| Escherichia_coli_XH140A | AFVX01000001 | non-ETEC |

**Supplemental Table 3. Accession numbers of Colonization factors and Virulence factors screened in this study.**

| target | type | accession |
|---|---|---|
| CFA/I | Colonization Factor | WP_000669509 |
| CS1 | Colonization Factor | CBL93542 |
| CS2 | Colonization Factor | CAA87761 |
| CS3 | Colonization Factor | CAA34820 |
| CS4 | Colonization Factor | AAK97135 |
| CS5 | Colonization Factor | CAA11820 |
| CS6 | Colonization Factor | AAC45093 |
| CS7 | Colonization Factor | AAK09045 |
| CS8 | Colonization Factor | BAA07174 |
| CS12 | Colonization Factor | AAK09047 |
| CS13 | Colonization Factor | CAA50789 |
| CS14 | Colonization Factor | CAA66122 |
| CS15 | Colonization Factor | CAA45906 |
| CS17 | Colonization Factor | AAS89777 |
| CS18 | Colonization Factor | AAB41914 |
| CS19 | Colonization Factor | AAQ19775 |
| CS20 | Colonization Factor | AAL31639 |
| CS21 | Colonization Factor | AAC33154 |
| CS22 | Colonization Factor | AAD30557 |
| CS23 | Colonization Factor | AFD29291 |
| PCF071 | Colonization Factor | AAS89773 |
| pcf_b02 | Colonization Factor | WP_001493678 |
| pcf_b03 | Colonization Factor | WP_001701908 |
| pcf_b04 | Colonization Factor | WP_001377911 |
| pcf_b05 | Colonization Factor | WP_004026086 |
| pcf_b06 | Colonization Factor | EMW44189 |
| pcf_b07 | Colonization Factor | WP_001741098 |
| *clyA* | Virulence Factor | AF240780_1 |
| *rns* | Virulence Factor | J04166 |
| *peaR* | Virulence Factor | WP_012000740 |
| *cexE* | Virulence Factor | WP_000759959 |
| *eaeH* | Virulence Factor | DQ109813 |
| *tibA* | Virulence Factor | AF109215 |
| *yghJ* | Virulence Factor | WP_001034464 |
| *eltA* | Virulence Factor | K01995 |
| *STP* | Virulence Factor | WP_001353651 |
| *STH* | Virulence Factor | AAA23990 |
| *eatA* | Virulence Factor | WP_001045019 |
| *etpA* | Virulence Factor | WP_001080112 |
| *irp2* | Virulence Factor | JQ868760 |
| *tia* | Virulence Factor | U20318 |
| *fyuA* | Virulence Factor | Z38064 |
| *leoA* | Virulence Factor | WP_000434627 |
| *astA* | Virulence Factor | WP_000477719 |
| *aatA* | Virulence Factor | WP_001394311 |
| *aatB* | Virulence Factor | WP_000776983 |
| *aatC* | Virulence Factor | WP_000593828 |
| *aatD* | Virulence Factor | WP_012131089 |

**Supplementary Table S4. Annotation of regions differentially conserved between different groups of genomes. Bold entries indicate a p value < 0.05**

| Group association | Average BSR value in group | Average BSR value not in group | FDR chi2 p-value | Accession | Annotation |
|---|---|---|---|---|---|
| **all symptomatic** | **0.80** | **0.40** | **0.01** | **gi\|758669266\|gb\|KIU72849.1\|** | **hypothetical protein C797_21044, partial [Bacillus thuringiensis Sbt003]** |
| **all symptomatic** | **0.70** | **0.33** | **0.02** | **gi\|535495231\|gb\|EQW34622.1\|** | **hypothetical protein G902_01788, partial [Escherichia coli UMEA 3052-1]** |
| **all symptomatic** | **0.71** | **0.31** | **0.02** | **gi\|754718663\|ref\|WP_042092166.1\|** | **tail assembly protein, partial [Escherichia coli]** |
| **all symptomatic** | **0.65** | **0.32** | **0.02** | **gi\|446401298\|ref\|WP_000479153.1\|** | **MULTISPECIES: phage minor tail protein G [Bacteria]** |
| **all symptomatic** | **0.68** | **0.33** | **0.03** | **gi\|585370479\|ref\|WP_024252050.1\|** | **hypothetical protein [Escherichia coli]** |
| **all symptomatic** | **0.72** | **0.35** | **0.03** | **gi\|477041355\|gb\|END25309.1\|** | **head decoration domain protein, partial [Escherichia coli P0302308.5]** |
| **all symptomatic** | **0.58** | **0.27** | **0.03** | **gi\|678158629\|gb\|KFV27073.1\|** | **phage tail fiber protein, partial [Escherichia coli]** |
| **all symptomatic** | **0.62** | **0.28** | **0.04** | **gi\|431045771\|gb\|ELD55994.1\|** | **hypothetical protein A17M_00003, partial [Escherichia coli KTE224]** |
| **all symptomatic** | **0.38** | **0.04** | **0.04** | **gi\|446522066\|ref\|WP_000599412.1\|** | **hypothetical protein [Escherichia coli]** |
| **all symptomatic** | **0.59** | **0.28** | **0.04** | **gi\|476130230\|gb\|EMV52443.1\|** | **phage major capsid E family protein [Escherichia coli 2867750]** |
| **all symptomatic** | **0.70** | **0.34** | **0.05** | **gi\|896039288\|ref\|WP_049094002.1\|** | **tail assembly protein, partial [Escherichia coli]** |
| **all symptomatic** | **0.67** | **0.30** | **0.05** | **gi\|446108993\|ref\|WP_000186848.1\|** | **MULTISPECIES: exonuclease [Enterobacteriaceae]** |
| **all symptomatic** | **0.58** | **0.24** | **0.05** | **gi\|430957671\|gb\|ELC76321.1\|** | **head decoration protein, partial [Escherichia coli KTE181]** |
| all symptomatic | 0.74 | 0.35 | 0.06 | gi\|535438559\|gb\|EQV79022.1\| | hypothetical protein G891_02112, partial [Escherichia coli KOEGE 68 (182a)] |
| all symptomatic | 0.68 | 0.34 | 0.07 | gi\|446121462\|ref\|WP_000199317.1\| | GntR family transcriptional regulator [Escherichia coli] |
| all symptomatic | 0.65 | 0.31 | 0.08 | gi\|320193379\|gb\|EFW68017.1\| | Phage major capsid protein [Escherichia coli WV_060327] |
| all symptomatic | 0.64 | 0.29 | 0.08 | gi\|446711531\|ref\|WP_000788877.1\| | MULTISPECIES: Replication protein 14 [Enterobacteriaceae] |
| all symptomatic | 0.36 | 0.03 | 0.12 | gi\|446337962\|ref\|WP_000415817.1\| | MULTISPECIES: hypothetical protein [Enterobacteriaceae] |
| all symptomatic | 0.68 | 0.34 | 0.13 | gi\|300402809\|gb\|EFJ86347.1\| | hypothetical protein HMPREF9536_03342 [Escherichia coli MS 84-1] |
| all symptomatic | 0.66 | 0.30 | 0.14 | gi\|446022992\|ref\|WP_000100847.1\| | MULTISPECIES: phage recombination protein Bet [Proteobacteria] |
| all symptomatic | 0.70 | 0.38 | 0.15 | gi\|445988719\|ref\|WP_000066574.1\| | chaperone protein FimC [Escherichia coli] |
| all symptomatic | 0.68 | 0.30 | 0.16 | gi\|446918235\|ref\|WP_000995491.1\| | host-nuclease inhibitor protein Gam [Escherichia coli] |
| all symptomatic | 0.71 | 0.39 | 0.18 | gi\|446116928\|ref\|WP_000194783.1\| | MULTISPECIES: endopeptidase [Enterobacteriaceae] |
| all symptomatic | 0.67 | 0.34 | 0.19 | gi\|446081020\|ref\|WP_000158875.1\| | MULTISPECIES: DNA-packaging protein FI [Enterobacteriaceae] |
| all symptomatic | 0.71 | 0.34 | 0.20 | gi\|485730284\|ref\|WP_001358225.1\| | MULTISPECIES: head decoration protein [Enterobacteriaceae] |
| all symptomatic | 0.61 | 0.30 | 0.24 | gi\|445985443\|ref\|WP_000063298.1\| | MULTISPECIES: phage capsid protein [Enterobacteriaceae] |
| all symptomatic | 0.72 | 0.39 | 0.31 | gi\|663434103\|sp\|P03735.2\|GT_LAMBD | RecName: Full=Tail assembly protein GT; AltName: Full=Gene product GT |
| all symptomatic | 0.70 | 0.36 | 0.33 | gi\|446605783\|ref\|WP_000683129.1\| | MULTISPECIES: tail protein [Enterobacteriaceae] |
| | | | | | |
| **alll asymptomatic** | **0.38** | **0.06** | **0.00** | **gi\|487484713\|ref\|WP_001699627.1\|** | **hypothetical protein [Escherichia coli]** |
| **alll asymptomatic** | **0.34** | **0.05** | **0.00** | **gi\|487653694\|ref\|WP_001748229.1\|** | **MULTISPECIES: addiction module antidote protein, HigA family [Enterobacteriaceae]** |
| **alll asymptomatic** | **0.38** | **0.06** | **0.00** | **gi\|485771319\|ref\|WP_001395310.1\|** | **membrane protein [Escherichia coli]** |
| **alll asymptomatic** | **0.46** | **0.14** | **0.00** | **gi\|449311601\|gb\|EMD01950.1\|** | **repB Plasmid Partition, partial [Escherichia coli O08]** |
| **alll asymptomatic** | **0.57** | **0.17** | **0.00** | **gi\|446537437\|ref\|WP_000614783.1\|** | **MULTISPECIES: hypothetical protein [Enterobacteriaceae]** |
| **alll asymptomatic** | **0.60** | **0.18** | **0.00** | **gi\|485770596\|ref\|WP_001394664.1\|** | **chromosome partitioning protein ParB [Escherichia coli]** |
| **alll asymptomatic** | **0.58** | **0.19** | **0.00** | **gi\|446057758\|ref\|WP_000135613.1\|** | **DNA invertase [Escherichia coli]** |
| **alll asymptomatic** | **0.43** | **0.11** | **0.00** | **FH07_00795** | **tRNA uridine 5-carboxymethylaminomethyl modification protein** |
| **alll asymptomatic** | **0.40** | **0.11** | **0.00** | **gi\|446344021\|ref\|WP_000421876.1\|** | **hypothetical protein [Escherichia coli]** |
| **alll asymptomatic** | **0.43** | **0.12** | **0.01** | **gi\|495141350\|ref\|WP_007866157.1\|** | **MULTISPECIES: hypothetical protein [Enterobacteriaceae]** |
| **alll asymptomatic** | **0.46** | **0.15** | **0.01** | **gi\|430890433\|gb\|ELC13070.1\|** | **hypothetical protein WCM_01852 [Escherichia coli KTE10]** |
| **alll asymptomatic** | **0.43** | **0.12** | **0.01** | **gi\|476309447\|gb\|EMX27231.1\|** | **phage integrase family protein [Escherichia coli MP021566.1]** |
| **alll asymptomatic** | **0.43** | **0.12** | **0.01** | **gi\|485699526\|ref\|WP_001332878.1\|** | **MULTISPECIES: hypothetical protein [Escherichia]** |
| **alll asymptomatic** | **0.42** | **0.12** | **0.01** | **gi\|693051054\|ref\|WP_032220188.1\|** | **hypothetical protein [Escherichia coli]** |
| **alll asymptomatic** | **0.43** | **0.12** | **0.01** | **gi\|446136142\|ref\|WP_000213997.1\|** | **hypothetical protein [Escherichia coli]** |
| **alll asymptomatic** | **0.43** | **0.12** | **0.01** | **gi\|476309446\|gb\|EMX27230.1\|** | **site-specific recombinase, phage integrase family [Escherichia coli MP021566.1]** |
| **alll asymptomatic** | **0.43** | **0.12** | **0.01** | **gi\|446576973\|ref\|WP_000654319.1\|** | **hypothetical protein [Escherichia coli]** |
| **alll asymptomatic** | **0.43** | **0.13** | **0.02** | **gi\|921493485\|emb\|CTW52716.1\|** | **Uncharacterised protein [Escherichia coli]** |
| **alll asymptomatic** | **0.51** | **0.18** | **0.02** | **gi\|485768778\|ref\|WP_001393114.1\|** | **initiator Replication family protein [Escherichia coli]** |
| **alll asymptomatic** | **0.43** | **0.13** | **0.02** | **gi\|485722304\|ref\|WP_001353500.1\|** | **MULTISPECIES: nipsnap [Escherichia]** |
| **alll asymptomatic** | **0.44** | **0.15** | **0.03** | **gi\|996070217\|ref\|WP_061089928.1\|** | **gluconate permease [Escherichia coli]** |
| **alll asymptomatic** | **0.43** | **0.12** | **0.03** | **gi\|446638713\|ref\|WP_000716059.1\|** | **MULTISPECIES: hypothetical protein [Enterobacteriaceae]** |
| **alll asymptomatic** | **0.43** | **0.14** | **0.03** | **gi\|485667986\|ref\|WP_001309159.1\|** | **MULTISPECIES: transcriptional regulator [Enterobacteriaceae]** |
| **alll asymptomatic** | **0.43** | **0.14** | **0.04** | **gi\|446921439\|ref\|WP_000998695.1\|** | **MULTISPECIES: gluconate 5-dehydrogenase [Enterobacteriaceae]** |
| **alll asymptomatic** | **0.64** | **0.29** | **0.04** | **gi\|446985088\|ref\|WP_001062344.1\|** | **integrase [Escherichia coli]** |
| **alll asymptomatic** | **0.56** | **0.22** | **0.04** | **gi\|446124307\|ref\|WP_000202162.1\|** | **hypothetical protein [Escherichia coli]** |
| alll asymptomatic | 0.48 | 0.18 | 0.05 | gi\|921695745\|emb\|CTV79180.1\| | putative integral membrane protein [Escherichia coli] |
| alll asymptomatic | 0.71 | 0.42 | 0.18 | gi\|446261995\|ref\|WP_000339850.1\| | hypothetical protein [Escherichia coli] |
| | | | | | |
| **ETEC** | **0.69** | **0.00** | **0.00** | **gi\|502953525\|ref\|WP_013188501.1\|** | **enterotoxin [Escherichia coli]** |
| **ETEC** | **0.64** | **0.00** | **0.00** | **gi\|476787230\|gb\|ENA88951.1\|** | **cholera enterotoxin subunit A [Escherichia coli 2862600]** |
| **ETEC** | **0.64** | **0.00** | **0.00** | **gi\|157021170\|gb\|ABV01318.1\|** | **LTA [Escherichia coli]** |
| **ETEC** | **0.64** | **0.00** | **0.00** | **gi\|502953503\|ref\|WP_013188479.1\|** | **enterotoxin [Escherichia coli]** |
| **ETEC** | **0.63** | **0.00** | **0.00** | **gi\|476147033\|gb\|EMV68856.1\|** | **hypothetical protein EC2866750_5055 [Escherichia coli 2866750]** |
| **ETEC** | **0.61** | **0.00** | **0.00** | **ECOESTA3A** | **estA3 gene econding heat-stable enterotoxin A3** |
| **ETEC** | **0.63** | **0.03** | **0.00** | **gi\|446967760\|ref\|WP_001045016.1\|** | **autotransporter [Escherichia coli]** |
| **ETEC** | **0.77** | **0.17** | **0.00** | **gi\|486165111\|ref\|WP_001528576.1\|** | **modulator protein [Escherichia coli]** |
| **ETEC** | **0.66** | **0.09** | **0.00** | **gi\|371597001\|gb\|EHN85826.1\|** | **hypothetical protein ESRG_02266 [Escherichia coli TA124]** |
| **ETEC** | **0.58** | **0.00** | **0.00** | **gi\|446268508\|ref\|WP_000346363.1\|** | **regulatory protein rns [Escherichia coli]** |
| **ETEC** | **0.61** | **0.04** | **0.00** | **gi\|693252299\|ref\|WP_032356369.1\|** | **invasion protein [Shigella flexneri]** |
| **ETEC** | **0.57** | **0.00** | **0.00** | **gi\|309706229\|emb\|CBJ04492.1\|** | **conserved hypothetical protein [Escherichia coli ETEC H10407]** |
| **ETEC** | **0.79** | **0.22** | **0.00** | **gi\|485727963\|ref\|WP_001356168.1\|** | **hypothetical protein [Escherichia coli]** |
| **ETEC** | **0.67** | **0.10** | **0.00** | **gi\|447194995\|ref\|WP_001272251.1\|** | **MULTISPECIES: hypothetical protein [Enterobacteriaceae]** |
| **ETEC** | **0.65** | **0.09** | **0.00** | **gi\|446508811\|ref\|WP_000586339.1\|** | **hypothetical protein [Escherichia coli]** |
| **ETEC** | **0.65** | **0.09** | **0.00** | **gi\|445941307\|ref\|WP_000019162.1\|** | **hypothetical protein [Escherichia coli]** |
| **ETEC** | **0.62** | **0.06** | **0.00** | **gi\|485652250\|ref\|WP_001297664.1\|** | **MULTISPECIES: holin [Enterobacteriaceae]** |
| **ETEC** | **0.71** | **0.15** | **0.00** | **gi\|253972543\|gb\|ACT38214.1\|** | **hypothetical protein ECB_00527 [Escherichia coli B str. REL606]** |
| **ETEC** | **0.71** | **0.15** | **0.00** | **gi\|763531332\|gb\|KIZ98396.1\|** | **hypothetical protein UH30_25090, partial [Escherichia coli]** |
| **ETEC** | **0.72** | **0.16** | **0.00** | **gi\|446812187\|ref\|WP_000889443.1\|** | **MULTISPECIES: hypothetical protein [Escherichia]** |
| **ETEC** | **0.89** | **0.34** | **0.00** | **gi\|331044156\|gb\|EGI16287.1\|** | **putative IS orf, partial [Escherichia coli M605]** |
| **ETEC** | **0.71** | **0.15** | **0.00** | **gi\|446551675\|ref\|WP_000629021.1\|** | **MULTISPECIES: hypothetical protein [Escherichia]** |
| **ETEC** | **0.57** | **0.03** | **0.00** | **gi\|332347856\|gb\|AEE60097.1\|** | **plasmid stability protein StbB [Escherichia coli UMNK88]** |
| **ETEC** | **0.54** | **0.00** | **0.00** | **gi\|446817573\|ref\|WP_000894829.1\|** | **hypothetical protein [Escherichia coli]** |
| **ETEC** | **0.54** | **0.00** | **0.00** | **gi\|447125825\|ref\|WP_001203081.1\|** | **hypothetical protein [Escherichia coli]** |
| **ETEC** | **0.54** | **0.00** | **0.00** | **gi\|378199984\|gb\|EHX60441.1\|** | **hypothetical protein ECDEC13D_3762 [Escherichia coli DEC13D]** |
| **ETEC** | **0.54** | **0.00** | **0.00** | **gi\|485768788\|ref\|WP_001393122.1\|** | **POTRA domain, ShlB-type family protein [Escherichia coli]** |
| **ETEC** | **0.57** | **0.03** | **0.00** | **gi\|447140849\|ref\|WP_001218105.1\|** | **MULTISPECIES: recombinase [Enterobacteriaceae]** |
| **ETEC** | **0.54** | **0.00** | **0.00** | **gi\|476698171\|gb\|ENA03359.1\|** | **hypothetical protein ECP02994382_2742 [Escherichia coli P0299438.2]** |
| **ETEC** | **0.75** | **0.21** | **0.00** | **gi\|446175012\|ref\|WP_000252867.1\|** | **transposase [Escherichia coli]** |
| **ETEC** | **0.53** | **0.00** | **0.00** | **gi\|485768877\|ref\|WP_001393184.1\|** | **glycosyl transferase [Escherichia coli]** |
| **ETEC** | **0.65** | **0.12** | **0.00** | **gi\|386226276\|gb\|EII48584.1\|** | **hypothetical protein EC23916_4858 [Escherichia coli 2.3916]** |
| **ETEC** | **0.53** | **0.00** | **0.00** | **gi\|502953525\|ref\|WP_013188501.1\|** | **enterotoxin [Escherichia coli]** |
| **ETEC** | **0.85** | **0.33** | **0.00** | **gi\|585353647\|ref\|WP_024235255.1\|** | **hypothetical protein, partial [Escherichia coli]** |
| **ETEC** | **0.83** | **0.33** | **0.00** | **gi\|485738727\|ref\|WP_001365958.1\|** | **hypothetical protein [Escherichia coli]** |

| | | | | | |
|---|---|---|---|---|---|
| ETEC | 0.84 | 0.34 | 0.00 | gi\|476916579\|gb\|ENC06472.1\| | hypothetical protein ECP02994386_5155 [Escherichia coli P0299438.6] |
| ETEC | 0.59 | 0.09 | 0.00 | gi\|485823023\|ref\|WP_001439368.1\| | insertion element family protein [Escherichia coli] |
| ETEC | 0.67 | 0.17 | 0.00 | gi\|446882614\|ref\|WP_000959870.1\| | recombinase [Escherichia coli] |
| ETEC | 0.56 | 0.06 | 0.00 | gi\|485775357\|ref\|WP_001398932.1\| | phage holin [Escherichia coli] |
| ETEC | 0.49 | 0.00 | 0.00 | gi\|446699637\|ref\|WP_000776983.1\| | ABC transporter [Escherichia coli] |
| ETEC | 0.55 | 0.06 | 0.00 | gi\|309701415\|emb\|CBJ00719.1\| | putative phage protein [Escherichia coli ETEC H10407] |
| ETEC | 0.75 | 0.26 | 0.00 | gi\|476301289\|gb\|EMX19181.1\| | hypothetical protein ECP03018671_2978 [Escherichia coli P0301867.1] |
| ETEC | 0.63 | 0.15 | 0.00 | gi\|485794512\|ref\|WP_001416057.1\| | transposase [Escherichia coli] |
| ETEC | 0.81 | 0.32 | 0.00 | gi\|485682829\|ref\|WP_001317493.1\| | MULTISPECIES: transposase [Enterobacteriaceae] |
| ETEC | 0.50 | 0.01 | 0.00 | coords: 26790-26945 | pETEC_74 plasmid region |
| ETEC | 0.49 | 0.00 | 0.00 | gi\|914254013\|ref\|WP_050561023.1\| | ABC transporter [Escherichia coli] |
| ETEC | 0.56 | 0.07 | 0.00 | gi\|446298810\|ref\|WP_000376665.1\| | MULTISPECIES: hypothetical protein [Enterobacteriaceae] |
| ETEC | 0.65 | 0.17 | 0.00 | gi\|727759186\|ref\|WP_033865056.1\| | hypothetical protein [Escherichia coli] |
| ETEC | 0.82 | 0.34 | 0.00 | gi\|446092831\|ref\|WP_000170686.1\| | hydrolase [Escherichia coli] |
| ETEC | 0.57 | 0.08 | 0.00 | gi\|477291003\|gb\|ENF62219.1\| | transposase family protein [Escherichia coli P0304816.8] |
| ETEC | 0.53 | 0.05 | 0.00 | gi\|486364177\|ref\|WP_001595202.1\| | MULTISPECIES: hypothetical protein [Escherichia] |
| ETEC | 0.50 | 0.01 | 0.00 | gi\|914278898\|ref\|WP_050562121.1\| | hypothetical protein, partial [Escherichia coli] |
| ETEC | 0.54 | 0.06 | 0.00 | gi\|485775358\|ref\|WP_001398933.1\| | hypothetical protein [Escherichia coli] |
| ETEC | 0.77 | 0.29 | 0.00 | gi\|446203792\|ref\|WP_000281647.1\| | MULTISPECIES: hypothetical protein [Escherichia] |
| ETEC | 0.54 | 0.06 | 0.00 | gi\|446415748\|ref\|WP_000493603.1\| | transposase [Escherichia coli] |
| ETEC | 0.49 | 0.02 | 0.00 | gi\|693196250\|ref\|WP_032318004.1\| | hypothetical protein, partial [Escherichia coli] |
| ETEC | 0.64 | 0.16 | 0.00 | gi\|446891640\|ref\|WP_000968896.1\| | protein stbB [Escherichia coli] |
| ETEC | 0.54 | 0.07 | 0.00 | gi\|476326681\|gb\|EMX44220.1\| | putative transposase domain protein [Escherichia coli MP020980.2] |
| ETEC | 0.47 | 0.00 | 0.00 | gi\|157076539\|gb\|ABV16252.1\| | putative membrane protein [Escherichia coli O139:H28 str. E24377A] |
| | | | | | |
| non-ETEC | 0.67 | 0.39 | 0.00 | gi\|446569212\|ref\|WP_000646558.1\| | hypothetical protein [Escherichia coli] |
| non-ETEC | 0.73 | 0.45 | 0.00 | gi\|481024513\|ref\|WP_001295635.1\| | MULTISPECIES: hypothetical protein [Escherichia] |
| non-ETEC | 0.41 | 0.13 | 0.00 | Coords: 1383259 - 1383152 | intergenic in E. coli VR50 |
| non-ETEC | 0.54 | 0.25 | 0.00 | gi\|446994520\|ref\|WP_001071776.1\| | hypothetical protein [Escherichia coli] |
| non-ETEC | 0.38 | 0.10 | 0.00 | gi\|485669906\|ref\|WP_001310834.1\| | MULTISPECIES: methyltransferase [Proteobacteria] |
| non-ETEC | 0.29 | 0.00 | 0.00 | gi\|446551385\|ref\|WP_000628731.1\| | EscE/YscE/SsaE family type III secretion system needle protein co-chaperone |
| non-ETEC | 0.29 | 0.00 | 0.00 | gi\|447196196\|ref\|WP_001273452.1\| | type III secretion system protein SepL [Escherichia coli] |
| non-ETEC | 0.29 | 0.00 | 0.00 | gi\|446925577\|ref\|WP_001002833.1\| | EscT/YscT/HrcT family type III secretion system export apparatus protein |
| non-ETEC | 0.29 | 0.00 | 0.00 | gi\|377955620\|gb\|EHV19176.1\| | hypothetical protein ECDEC4E_0548 [Escherichia coli DEC4E] |
| non-ETEC | 0.29 | 0.00 | 0.00 | gi\|446009612\|ref\|WP_000087467.1\| | CesD/SycD/LcrH family type III secretion system chaperone |
| non-ETEC | 0.29 | 0.00 | 0.00 | gi\|447214437\|ref\|WP_001291693.1\| | EscU/YscU/HrcU family type III secretion system export apparatus switch protein |
| non-ETEC | 0.29 | 0.00 | 0.00 | gi\|446960569\|ref\|WP_001037825.1\| | EscV/YscV/HrcV family type III secretion system export apparatus protein |
| non-ETEC | 0.29 | 0.00 | 0.00 | gi\|974633843\|ref\|WP_059220517.1\| | EscC/YscC/HrcC family type III secretion system outer membrane ring protein |
| non-ETEC | 0.29 | 0.00 | 0.00 | gi\|446366323\|ref\|WP_000444178.1\| | hypothetical protein [Escherichia coli] |
| non-ETEC | 0.30 | 0.01 | 0.00 | gi\|447210241\|ref\|WP_001287497.1\| | MULTISPECIES: N(6)-hydroxylysine O-acetyltransferase [Escherichia] |
| non-ETEC | 0.29 | 0.00 | 0.00 | gi\|290764943\|gb\|ADD58904.1\| | EscS [Escherichia coli O55:H7 str. CB9615] |
| non-ETEC | 0.29 | 0.01 | 0.00 | gi\|806460057\|ref\|WP_046125291.1\| | aerobactin synthase IucA, partial [Escherichia coli] |
| non-ETEC | 0.77 | 0.48 | 0.00 | gi\|476758914\|gb\|ENA62521.1\| | hypothetical protein EC178900_2124 [Escherichia coli 178900] |
| non-ETEC | 0.29 | 0.00 | 0.00 | gi\|12518477\|gb\|AAG58847.1\|AE005597_5 | escR [Escherichia coli O157:H7 str. EDL933] |
| non-ETEC | 0.30 | 0.01 | 0.00 | gi\|903858119\|emb\|CSF78069.1\| | N(6)-hydroxylysine O-acetyltransferase%2C aerobactin biosynthetic [Shigella sonnei] |
| non-ETEC | 0.30 | 0.01 | 0.00 | gi\|446672793\|ref\|WP_000750139.1\| | MULTISPECIES: lysine 6-monooxygenase [Escherichia] |
| non-ETEC | 0.30 | 0.01 | 0.00 | gi\|446938453\|ref\|WP_001015709.1\| | MULTISPECIES: aerobactin synthase IucC [Escherichia] |
| non-ETEC | 0.30 | 0.00 | 0.00 | gi\|485669336\|ref\|WP_001310358.1\| | LEE-encoded type III secretion system factor [Escherichia coli] |
| non-ETEC | 0.57 | 0.28 | 0.00 | gi\|486361791\|ref\|WP_001594108.1\| | hypothetical protein [Escherichia coli] |
| non-ETEC | 0.81 | 0.51 | 0.00 | gi\|447015715\|ref\|WP_001092971.1\| | MULTISPECIES: lysozyme from lambdoid prophage Qin [Proteobacteria] |
| non-ETEC | 0.31 | 0.02 | 0.00 | gi\|607756438\|gb\|EZE37703.1\| | hypothetical protein BX19_01100, partial [Escherichia coli O121:H19 str. 2009C-4050] |
| non-ETEC | 0.30 | 0.00 | 0.00 | gi\|446020936\|ref\|WP_000098791.1\| | tir chaperone [Escherichia coli] |
| non-ETEC | 0.91 | 0.62 | 0.00 | gi\|487505501\|ref\|WP_001705358.1\| | L-fucose-proton symporter domain protein [Escherichia coli] |
| non-ETEC | 0.52 | 0.23 | 0.00 | gi\|446548285\|ref\|WP_000625631.1\| | MULTISPECIES: phosphodiesterase YaeI [Proteobacteria] |
| non-ETEC | 0.30 | 0.00 | 0.00 | gi\|13447697\|gb\|AAK26696.1\|AF200363_3 | Ler [Escherichia coli] |
| non-ETEC | 0.77 | 0.47 | 0.00 | gi\|535679400\|gb\|EQY14887.1\| | hypothetical protein G943_03937 [Escherichia coli UMEA 3212-1] |
| non-ETEC | 0.37 | 0.07 | 0.00 | gi\|446951598\|ref\|WP_001028854.1\| | MULTISPECIES: serine/threonine protein phosphatase [Escherichia] |
| non-ETEC | 0.56 | 0.26 | 0.00 | gi\|325499894\|gb\|EGC97753.1\| | hypothetical protein ECD227_3991 [Escherichia fergusonii ECD227] |
| non-ETEC | 0.30 | 0.00 | 0.00 | gi\|444584541\|gb\|ELV60175.1\| | hypothetical protein EC991775_0865 [Escherichia coli 99.1775] |
| non-ETEC | 0.31 | 0.01 | 0.00 | gi\|446157581\|ref\|WP_000235436.1\| | MULTISPECIES: terminase [Proteobacteria] |
| non-ETEC | 0.30 | 0.00 | 0.00 | gi\|446976584\|ref\|WP_001053840.1\| | MULTISPECIES: EscF/YscF/HrpA family type III secretion system needle major subunit |
| non-ETEC | 0.30 | 0.00 | 0.00 | gi\|446150730\|ref\|WP_000228585.1\| | hypothetical protein [Escherichia coli] |
| non-ETEC | 0.56 | 0.26 | 0.00 | gi\|115515832\|gb\|ABJ03907.1\| | conserved hypothetical protein [Escherichia coli APEC O1] |
| non-ETEC | 0.32 | 0.02 | 0.00 | gi\|394390844\|gb\|EJE67781.1\| | terminase large subunit, partial [Escherichia coli O26:H11 str. CVM10224] |
| non-ETEC | 0.77 | 0.46 | 0.00 | gi\|938524511\|gb\|KPQ49112.1\| | Uncharacterized protein TW10598_3397 [Escherichia coli TW10598] |
| non-ETEC | 0.67 | 0.35 | 0.00 | gi\|26109426\|gb\|AAN81629.1\|AE016765_31 | Cold shock-like protein cspI [Escherichia coli CFT073] |
| non-ETEC | 0.45 | 0.13 | 0.00 | gi\|485656937\|ref\|WP_001301033.1\| | MULTISPECIES: protein FlxA [Proteobacteria] |
| non-ETEC | 0.34 | 0.01 | 0.00 | gi\|606793145\|gb\|EYY33044.1\| | hypothetical protein BX84_17030 [Escherichia coli O121:H19 str. 2010C-4989] |
| non-ETEC | 0.33 | 0.00 | 0.00 | gi\|408582104\|gb\|EKK57351.1\| | hypothetical protein EC82524_2797, partial [Escherichia coli 8.2524] |
| non-ETEC | 0.59 | 0.25 | 0.00 | gi\|446903738\|ref\|WP_000980994.1\| | MULTISPECIES: Qin prophage; uncharacterized protein [Proteobacteria] |
| non-ETEC | 0.39 | 0.04 | 0.00 | gi\|650101289\|gb\|KDU22111.1\| | TAP-like family protein [Escherichia coli 3-267-03_S4_C2] |
| non-ETEC | 0.70 | 0.35 | 0.00 | gi\|445988629\|ref\|WP_000066484.1\| | MULTISPECIES: cold-shock protein CspB [Proteobacteria] |
| non-ETEC | 0.40 | 0.04 | 0.00 | gi\|447149119\|ref\|WP_001226375.1\| | MULTISPECIES: hydrolase [Escherichia] |
| non-ETEC | 0.55 | 0.16 | 0.00 | gi\|693257255\|ref\|WP_032360235.1\| | hypothetical protein [Escherichia coli] |
| non-ETEC | 0.55 | 0.16 | 0.00 | gi\|446265012\|ref\|WP_000342867.1\| | MULTISPECIES: hypothetical protein [Proteobacteria] |
| non-ETEC | 0.53 | 0.12 | 0.00 | gi\|345369821\|gb\|EGX01801.1\| | hypothetical protein ECSTECH18_5093 [Escherichia coli STEC_H.1.8] |
| non-ETEC | 0.62 | 0.22 | 0.00 | gi\|446969849\|ref\|WP_001047105.1\| | MULTISPECIES: antitermination protein [Enterobacteriaceae] |
| non-ETEC | 0.53 | 0.12 | 0.00 | gi\|378017415\|gb\|EHV80288.1\| | hypothetical protein ECDEC7A_1760 [Escherichia coli DEC7A] |
| non-ETEC | 0.55 | 0.12 | 0.00 | gi\|260449321\|gb\|ACX39743.1\| | hypothetical protein EcDH1_2087 [Escherichia coli DH1] |
| non-ETEC | 0.72 | 0.29 | 0.00 | gi\|812042635\|emb\|CRH31059.1\| | hypothetical protein BN1182_CK_00120 [Pantoea ananatis] |
| non-ETEC | 0.56 | 0.12 | 0.00 | gi\|446383935\|ref\|WP_000461790.1\| | hypothetical protein [Escherichia coli] |
| non-ETEC | 0.57 | 0.13 | 0.00 | gi\|385713391\|gb\|EIG50324.1\| | hypothetical protein ESTG_00016 [Escherichia coli B799] |
| non-ETEC | 0.77 | 0.33 | 0.00 | Coords: 4598974 - 4599033 | Present in E. coli O7:K1 str. CD10 |
| non-ETEC | 0.68 | 0.22 | 0.00 | gi\|545247112\|ref\|WP_021545569.1\| | phage antitermination protein [Escherichia coli] |
| | | | | | |
| Group 1 symptomati | 1.00 | 0.00 | 0.00 | gi\|446548321\|ref\|WP_000625667.1\| | MULTISPECIES: hypothetical protein [Enterobacteriaceae] |
| Group 1 symptomati | 1.00 | 0.00 | 0.00 | gi\|446052268\|ref\|WP_000130123.1\| | MULTISPECIES: hypothetical protein [Escherichia] |
| Group 1 symptomati | 1.00 | 0.00 | 0.00 | gi\|445927697\|ref\|WP_000005552.1\| | MULTISPECIES: DNA-binding protein [Proteobacteria] |
| Group 1 symptomati | 1.00 | 0.00 | 0.00 | gi\|693005527\|ref\|WP_032190252.1\| | hypothetical protein [Escherichia coli] |
| Group 1 symptomati | 1.00 | 0.00 | 0.00 | gi\|446759498\|ref\|WP_000836754.1\| | MULTISPECIES: hypothetical protein [Escherichia] |
| Group 1 symptomati | 1.00 | 0.00 | 0.04 | gi\|446270625\|ref\|WP_000348480.1\| | threonine dehydrogenase [Shigella sonnei] |
| Group 1 symptomati | 0.97 | 0.00 | 0.00 | gi\|446262973\|ref\|WP_000340828.1\| | hypothetical protein [Escherichia coli] |
| | | | | | |
| Group1 asymptomat | 1.00 | 0.00 | 0.00 | gi\|487679144\|ref\|WP_001764021.1\| | phage stabilization family protein [Escherichia coli] |
| Group1 asymptomat | 1.00 | 0.00 | 0.00 | gi\|487490747\|ref\|WP_001701215.1\| | phage terminase large subunit PBSX family [Escherichia coli] |
| Group1 asymptomat | 1.00 | 0.00 | 0.00 | gi\|487490656\|ref\|WP_001701197.1\| | regulatory protein mnt [Escherichia coli] |
| Group1 asymptomat | 1.00 | 0.00 | 0.00 | gi\|693100130\|ref\|WP_032255648.1\| | hypothetical protein [Escherichia coli] |

| | | | | | |
|---|---|---|---|---|---|
| **Group1 asymptomat** | **1.00** | **0.00** | **0.00** | **gi\|446297782\|ref\|WP_000375637.1\|** | **MULTISPECIES: hypothetical protein [Enterobacteriaceae]** |
| **Group1 asymptomat** | **1.00** | **0.00** | **0.00** | **gi\|487490749\|ref\|WP_001701217.1\|** | **DNA transfer protein gp20 [Escherichia coli]** |
| **Group1 asymptomat** | **1.00** | **0.00** | **0.00** | **gi\|446706388\|ref\|WP_000783734.1\|** | **MULTISPECIES: phage holin, lambda family [Enterobacteriaceae]** |
| | | | | | |
| Group2 symptomatic | 0.67 | 0.00 | 0.57 | gi\|485772405\|ref\|WP_001396300.1\| | MULTISPECIES: tniQ family protein [Enterobacteriaceae] |
| Group2 symptomatic | 0.67 | 0.00 | 0.57 | gi\|446971924\|ref\|WP_001049180.1\| | MULTISPECIES: transposase [Proteobacteria] |
| Group2 symptomatic | 0.67 | 0.02 | 0.60 | gi\|446184568\|ref\|WP_000262423.1\| | MULTISPECIES: hypothetical protein [Enterobacteriaceae] |
| Group2 symptomatic | 0.73 | 0.09 | 0.30 | gi\|447019960\|ref\|WP_001097216.1\| | MULTISPECIES: transcriptional regulator [Proteobacteria] |
| Group2 symptomatic | 0.66 | 0.05 | 0.64 | gi\|763494632\|gb\|KIZ63970.1\| | Tn7 transposition protein B, partial [Escherichia coli] |
| Group2 symptomatic | 0.92 | 0.33 | 0.13 | gi\|446369064\|ref\|WP_000446919.1\| | hypothetical protein [Escherichia coli] |
| | | | | | |
| Group2 asymptomatic | 1 | 0.50 | 0.77 | gi\|998227648\|ref\|WP_061093255.1\| | DNA methylase [Escherichia coli] |
| Group2 asymptomatic | 1 | 0.49 | 0.64 | gi\|485774630\|ref\|WP_001398340.1\| | DEAD/DEAH box helicase [Escherichia coli] |
| Group2 asymptomatic | 1 | 0.48 | 0.64 | gi\|447002552\|ref\|WP_001079808.1\| | MULTISPECIES: hypothetical protein [Enterobacteriaceae] |
| Group2 asymptomatic | 1 | 0.47 | 0.77 | gi\|485771346\|ref\|WP_001395333.1\| | DEAD/DEAH box helicase [Escherichia coli] |
| Group2 asymptomatic | 1 | 0.42 | 0.73 | gi\|378179931\|gb\|EHX40636.1\| | shufflon protein D [Escherichia coli DEC12E] |
